# All SNPs Are Not Created Equal: Genome-Wide Association Studies Reveal a Consistent Pattern of Enrichment among Functionally Annotated SNPs

**DOI:** 10.1371/journal.pgen.1003449

**Published:** 2013-04-25

**Authors:** Andrew J. Schork, Wesley K. Thompson, Phillip Pham, Ali Torkamani, J. Cooper Roddey, Patrick F. Sullivan, John R. Kelsoe, Michael C. O'Donovan, Helena Furberg, The Tobacco and Genetics Consortium, The Bipolar Disorder Psychiatric Genomics Consortium, The Schizophrenia Psychiatric Genomics Consortium, Nicholas J. Schork, Ole A. Andreassen, Anders M. Dale

**Affiliations:** 1 Cognitive Sciences Graduate Program, University of California San Diego, La Jolla, California, United States of America; 2 Center for Human Development, University of California San Diego, La Jolla, California, United States of America; 3 Multimodal Imaging Laboratory, University of California San Diego, La Jolla, California, United States of America; 4 Department of Psychiatry, University of California San Diego, La Jolla, California, United States of America; 5 Scripps Health, La Jolla, California, United States of America; 6 The Scripps Translational Science Institute, The Scripps Research Institute, La Jolla, California, United States of America; 7 Department of Molecular and Experimental Medicine, The Scripps Research Institute, La Jolla, California, United States of America; 8 Department of Genetics, University of North Carolina, Chapel Hill, North Carolina, United States of America; 9 MRC Centre for Neuropsychiatric Genetics and Genomics, School of Medicine, Cardiff University, Cardiff, United Kingdom; 10 Department of Epidemiology and Biostatistics, Memorial Sloan Kettering Cancer Center, New York, New York, United States of America; 11 Institute of Clinical Medicine, University of Oslo, Oslo, Norway; 12 Division of Mental Health and Addiction, Oslo University Hospital, Oslo, Norway; 13 Department of Cognitive Sciences, University of California San Diego, La Jolla, California, United States of America; 14 Department of Radiology, University of California San Diego, La Jolla, California, United States of America; 15 Department of Neurosciences, University of California San Diego, La Jolla, California, United States of America; Georgia Institute of Technology, United States of America

## Abstract

Recent results indicate that genome-wide association studies (GWAS) have the potential to explain much of the heritability of common complex phenotypes, but methods are lacking to reliably identify the remaining associated single nucleotide polymorphisms (SNPs). We applied stratified False Discovery Rate (sFDR) methods to leverage genic enrichment in GWAS summary statistics data to uncover new loci likely to replicate in independent samples. Specifically, we use linkage disequilibrium-weighted annotations for each SNP in combination with nominal p-values to estimate the True Discovery Rate (TDR = 1−FDR) for strata determined by different genic categories. We show a consistent pattern of enrichment of polygenic effects in specific annotation categories across diverse phenotypes, with the greatest enrichment for SNPs tagging regulatory and coding genic elements, little enrichment in introns, and negative enrichment for intergenic SNPs. Stratified enrichment directly leads to increased TDR for a given p-value, mirrored by increased replication rates in independent samples. We show this in independent Crohn's disease GWAS, where we find a hundredfold variation in replication rate across genic categories. Applying a well-established sFDR methodology we demonstrate the utility of stratification for improving power of GWAS in complex phenotypes, with increased rejection rates from 20% in height to 300% in schizophrenia with traditional FDR and sFDR both fixed at 0.05. Our analyses demonstrate an inherent stratification among GWAS SNPs with important conceptual implications that can be leveraged by statistical methods to improve the discovery of loci.

## Author Summary

Modern genome-wide association studies (GWAS) have failed to identify large portions of the genetic basis of common, complex traits. Recent work suggested this could be because many genetic variants, each with individually small effects, compose their genetic architecture, limiting the power of GWAS. Moreover, these variants appear more abundantly in and near genes. Using genome annotations, summary statistics from several of the largest GWAS, and established statistical methods for quantifying distributions of test statistics, we show a consistency across studies. Namely, we show that, across all assessed traits, the test statistics resulting from SNPs that are related to the 5′ UTR of genes show the largest abundance of associations, while SNPs related to exons and the 3′UTR are also enriched. SNPs related to introns are only moderately enriched, and intergenic SNPs show a depletion of associations relative to the average SNP. This enrichment corresponds directly to increased replication across independent samples and can be incorporated *a priori* into statistical methods to improve discovery and prediction. Our results contribute to on-going debates about the functional nature of the genetic architecture of complex traits and point to avenues for leveraging existing GWAS data for discovery in future GWA and sequencing studies.

## Introduction

Complex traits are generally influenced by many genes with small individual effects [1]. This ‘polygenic’ architecture has been difficult to characterize. While Genome-wide association studies (GWAS) [2] have successfully identified thousands of trait-associated single nucleotide polymorphisms (SNPs) [3], even when considered in aggregate, these SNPs explain small portions of the trait heritability [4]. Recent results indicate that GWAS have the potential to explain much of the heritability of common complex phenotypes [5], [6], and more SNPs are likely to be identified in larger samples [7]. However, there are few methods available for identifying more of the SNPs likely to be associated with phenotypes without increasing the sample size, as recognized by recent European and US calls for new statistical genetics methods. The crucial issue is that for most complex traits a large number of SNPs have too small an effect to pass standard GWAS significance thresholds given current sample sizes. We present results suggesting new analytical approaches for GWAS will uncover more of the polygenic effects in complex disorders and traits. We hypothesize that all SNPs in a GWAS are not exchangeable, but come from pre-determinable categories with different distributions of effects. This implies that some categories of SNPs are enriched, i.e. are more likely to be associated with a phenotype than others. This information can be used to calculate the category-specific True Discovery Rate (TDR), or the expected proportion of correctly rejected null hypotheses [8]. SNPs from enriched SNP categories will have an increased TDR for a given effect size, or equivalently, for a given nominal p-value. Stratified False Discovery Rate (sFDR) methods [9] provide an established framework for demonstrating the utility of using enriched genic categories to increase power to discover SNPs likely to replicate in independent samples. Previous work has applied sFDR and related methods to GWAS data stratified by candidate regions determined through prior linkage analysis and/or candidate gene studies [10]–[12] and specific biological pathways related to disease etiology [13]. Others have considered stratification by genome annotations in linkage analysis [14] and Bayesian association analyses [15], demonstrating the utility of this approach for improving power and FDR based discovery where reliable, pre-determinable strata exist.

It has been suggested that variation in and around genes harbors more polygenic effects [6], [16]. However, the particular gene elements (i.e., intron, exon, UTRs) containing these variants and the distribution of effect sizes in GWAS have been left to extrapolation and speculation. Further, SNPs in and around genes have been shown to explain more variation [6] and replicate at higher rates [16] than intergenic SNPs. These studies, however, did not parse genic regions down to specific genic elements. We here hypothesize that SNPs in regulatory and coding elements of protein coding genes will show an enrichment of polygenic effects relative to intronic and intergenic SNPs which will be reflected in an increased estimated TDR and empirically confirmed through improved replication rate across independent samples.

The association signal of a SNP tested in GWAS is a surrogate for, or ‘tags,’ the potential effects of many other variants. Thus, any of a number of ‘tagged’ variants could underlie the observed association signal. Focusing on the tag SNPs only, without systematically capturing the underlying causal variants within a ‘tagged’ linkage block, limits the functional inferences that can be drawn from GWAS. By incorporating the correlation between SNPs (linkage disequilibrium; LD) we expect a stronger and more consistent differentiation of enrichment among genic annotation categories. In the current study, we use an LD-weighted scoring algorithm that allows quantification of the properties of multi-locus LD structure implicitly captured by each tag SNP to our enrichment analysis. These categories can be leveraged to create strata for established sFDR approaches.

We employ a model free strategy to identify enriched strata among phenotypes based on GWAS summary statistics. We first calculate the relative enrichment in different genic elements, using the category-specific empirical cumulative distribution function (cdf) of the nominal p-values after controlling for estimated genomic inflation. For each nominal p-value threshold an estimate of the category-specific TDR = 1−FDR is obtained from these empirical cdfs. This analysis is implemented on summary p-values from ten published GWAS meta-analyses studying 14 phenotypes. We then use the sub-study GWAS in Crohn's disease to test if the estimated increased TDR translates to improved replication rates, showing that for a given replication rate the nominal p-value threshold is 100 times larger for the most enriched genic category compared to the intergenic category. Finally, using an established sFDR framework we demonstrate the utility of leveraging enriched categories for improving power to detect SNPs likely to replicate, i.e., to reject more null hypotheses for a fixed FDR.

## Results

### LD-Based Enrichment of Genic Elements in Height

Under multiple testing paradigms such as GWAS, quantitative estimates of likely true associations can be estimated from the distributions of summary statistics [17], [18]. A common method for visualizing the enrichment of statistical association relative to that expected under the global null hypothesis is through Q-Q plots of the nominal p-values resulting from GWAS. Under the global null hypothesis the theoretical distribution is uniform on the interval [0,1]. Thus, the usual Q-Q curve has as the y-coordinate the nominal p-value, denoted by “p”, and the x-coordinate the value of the empirical cdf at p, which we denote by “q”. As is common in GWAS, we instead plot −log_10_ p against the −log_10_ q to emphasize tail probabilities of the theoretical and empirical distributions. In such plots, enrichment results in a leftward shift in the Q-Q curve, corresponding to a larger fraction of SNPs with nominal −log_10_ p-value greater than or equal to a given threshold (see Material and Methods).

**Figure 1:**
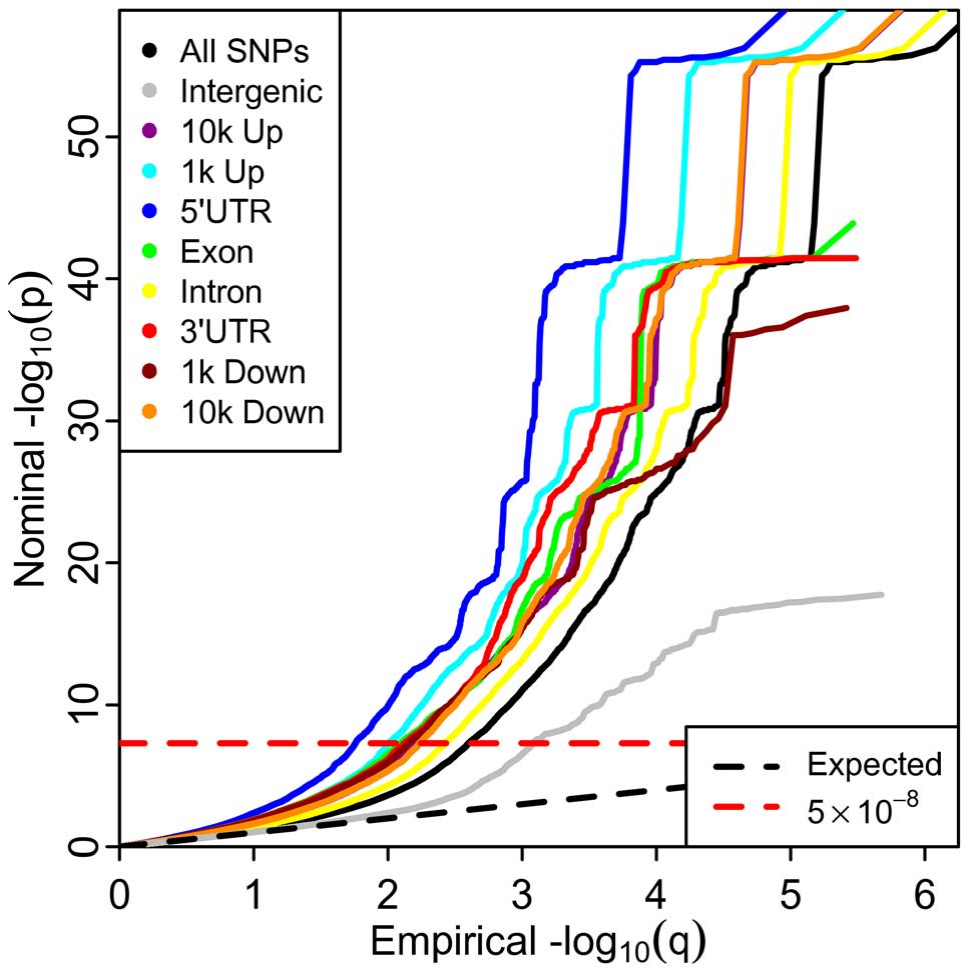
Stratified Q-Q plot for height shows enrichment by annotation categories using Linkage-Disequilibrium (LD)-weighted scores. Genic annotation categories were: 1) 10,000 to 1,001 base pairs upstream (10 k Up); 2) 1,000 to 1 base pair upstream (1 k Up); 3) 5′ untranslated region (5′UTR); 4) Exon; 5) Intron; 6) 3′ untranslated region (3′UTR); 7) 1 to 1,000 base pairs downstream (1 k Down); 8) 1,001 to 10,000 base pairs downstream (10 k Down). Q-Q plot of height with non-LD weighted category scores are shown in Figure S3.

The stratified Q-Q plot for height (Figure 1) shows a clear variation in enrichment across genic annotation categories. The separation between the curves for different categories is enhanced when using LD-weighted genic annotation categories in comparison to non LD-weighted positional categories (Figure S3). The parallel shape of these curves is likely caused by the significant but imperfect correlation among categories due to the non-exclusive nature of the annotation scoring (Figure S2).

An earlier departure from the null line (leftward shift) suggests a greater proportion of true associations, for a given nominal p-value. The divergence of the curves for different categories implies that the proportion of non-null effects varies considerably among annotation categories of genic elements. For example, the proportion of SNPs in the 5′UTR category reaching a significance level of −log_10_(p)>10 is roughly 10 times greater than for all SNPs and 50–100 times greater than for intergenic SNPs.

### Polygenic Enrichment across Diverse Phenotypes

**Figure 2:**
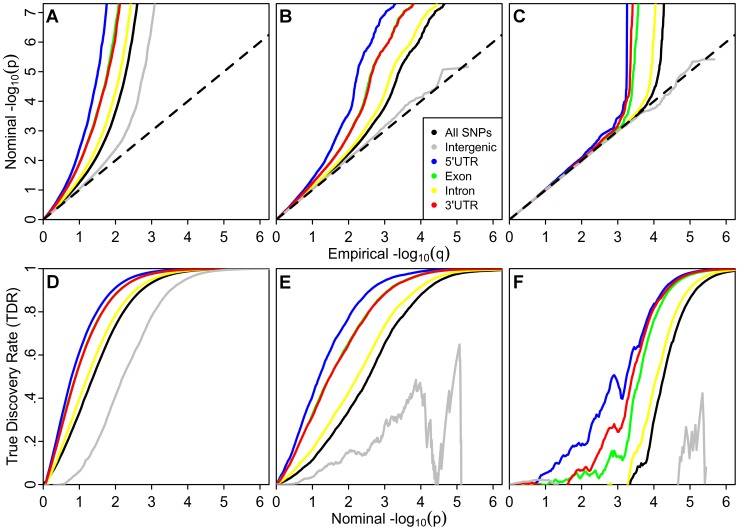
Stratified Q-Q plots and true discovery rates show consistency of enrichment. *Upper panel:* Stratified Q-Q plots illustrating consistent enrichment of genic annotation categories across diverse phenotypes: (A) Height, (B) Schizophrenia (SCZ), and (C) Cigarettes per Day (CPD). All figures are corrected for inflation using intergenic inflation control. Only nominal p-values below the standard genome-wide significance threshold (p<5×10^−8^) are shown. *Lower panel:* Stratified True Discovery Rate (TDR) plots illustrating the increase in TDR associated with increased enrichment in (D) Height, (E) SCZ and (F) CPD. Genic annotation categories were: 5′ untranslated region (5′UTR), Exon, Intron, 3′ untranslated region (3′UTR), All SNPs, in addition to Intergenic.

Recently Yang et al [19] demonstrated that an abundance of low p-values beyond what is expected under null hypotheses in GWAS, but not necessarily reaching stringent multiple comparison thresholds, often attributed to ‘spurious inflation,’ is also consistent with an enrichment of true ‘polygenic’ effects [19]. The prevalence of enrichment below the established genome-wide significance threshold of p<5×10^−8^ (−log_10_(p)>7.3;) in height (Figure 2A) is consistent with their hypotheses and strongly suggests that current GWAS do not capture all of the additive ‘tagged variance’ in this phenotype. Importantly, this enrichment varies across genic annotation categories.

The enrichment patterns among annotation categories are consistent across phenotypes, including schizophrenia (SCZ) and tobacco smoking (cigarettes per day, CPD; Figure 2B–2C). The stratified Q-Q plots for height, SCZ and CPD each demonstrate the largest enrichment for tag SNPs in LD with 5′UTR, and exonic variation, showing nearly tenfold increases in terms of the proportion of p-values expected below a given threshold under the null hypothesis. SNPs that tag intergenic regions show nearly tenfold depletions in comparison to all tag SNPs, although not when compared to the expected null. SNPs tagging intronic variation show minimal enrichment over all tag SNPs, despite making up the largest proportion of genic SNPs (Table S3). The pattern is consistent for all phenotypes considered (data not shown). Given the log scale of the Q-Q plots, 90% of SNPs fall between 0 and 1 and 99% fall between 0 and 2 on the horizontal axis, and thus it is clear that a majority of enriched SNPs have p-values that do not reach genome-wide significance.

We computed significance values for the curves for each annotation category relative to those for intergenic SNPs, using a two-sample Kolmogorov-Smirnov Test. The enrichment for height was highly significant for all categories when compared with the intergenic category, with all p-values less than 2.2×10^−16^. Nearly every genic category was also significantly enriched for each other phenotype (Table S5).

While the pattern of enrichment is consistent, the shape of the curves varies across phenotypes. In particular, the point at which the curves deviate from the expected null line occurs earliest for height, followed by SCZ, and finally CPD (Figure 2A–2C), consistent with different proportions of SNPs that are likely associated with each trait (i.e., different levels of ‘polygenicity’). These findings are consistent with results obtained using an established mixture-modeling framework [17] (Text S1 and Figures S8, S9, S10, S17, S18, S19).

### Intergenic Genomic Control

The relative absence of enrichment in intergenic SNPs as we have defined them, suggests minimal inflation due to polygenic effects and a more robust estimate of the global null. This fact can be exploited for better estimation of variance inflation due to stratification [20] that is minimally confounded by true polygenic effects [19]. We confined the estimation of the genomic inflation factor [20], λ_GC_, to only intergenic SNPs (Table S4) and adjusted summary statistics for all phenotypes according to this “intergenic inflation control” procedure. The stratified Q-Q plots for height with and without intergenic inflation control are shown in Figure S4.

### Category-Specific True Discovery Rate

Since specific tag SNP categories are significantly more likely to be associated with common phenotypes, while intergenic ones are less likely, all tag SNPs should not be treated as exchangeable. Variation in enrichment across diverse genic categories is expected to be associated with corresponding variation in TDR for a given nominal p-value threshold. A conservative estimate of the TDR for each nominal p-value is equivalent to 1−(p/q) as plotted on the Q-Q plots (see Online Methods). This relationship is shown for height, SCZ and CPD (Figure 2D–2E). Similar category-specific TDR plots were calculated for each of the 14 phenotypes (data not shown). For a given TDR the corresponding estimated nominal p-value threshold varies with a factor of 100 from the most enriched genic category to the intergenic category, and the pattern is consistent across phenotypes. Since TDR is theoretically related to predicted replication rate, it is expected that for a given p-value threshold the replication rate will be higher for SNPs in genic categories with high TDR. The high estimates of TDR at significance levels below genome-wide significance is consistent with recent work in Schizophrenia that demonstrates a high proportion of likely true associations at reduced thresholds, but without the needed power to reach genome-wide significance [21].

### Quantification of Enrichment

**Figure 3:**
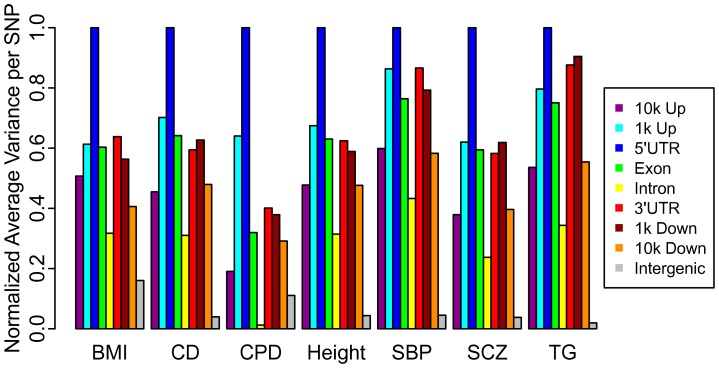
Categorical enrichment for seven diverse phenotypes. The relative pattern of enrichment, as measured by the mean (z-score^2^−1) after intergenic inflation control, of LD-weighted genic annotation categories remain consistent. Results for all phenotypes are shown in Figure S5, Table S6.

While the TDR provides a quantification of enrichment for a given nominal p-value threshold (equivalently, SNP z-score threshold), we also provide a single number quantification of enrichment for each LD-weighted annotation category within each phenotype, computed as the sample mean (z^2^)−1. The sample mean, taken over all SNPs in a given category, provides an estimate of the variance due to null and non-null SNPs; by subtracting one we obtain a conservative estimate of the variance in effect sizes attributable to non-null SNPs alone. Both TDR and mean (z^2^)−1 are justified based on a standard mixture model formulation (see Text S1). These enrichment scores, normalized by the maximum value across categories within each phenotype, are presented in Figure 3. The 5′UTR annotation category was the most enriched category across all fourteen phenotypes (Table S6). Additionally, the exon category is consistently more enriched than the intron category.

Categories where each SNP tags more reference SNPs on average or represents a larger total amount of LD could spuriously appear enriched. We do note categorical differences in the number of SNPs and total summed LD captured by each SNP (Tables S7 and S8) but multiple regression analyses show the effect of these variables is negligible and independent categorical effects persist (Table S10) despite the significant correlation among categories (Figure S2). Likewise, systematic deviations in minor allele frequency (MAF) across categories could bias annotation category effects as MAF acts multiplicatively with effect size to explain variance. We found minimal categorical stratification for MAF that is inconsistent with this effect driving our enrichment findings (Table S9 and Figure S6). To further address the possibility that some of the differential enrichment of categories could be due to category-specific genomic inflation from the above factors, we performed null-GWAS simulations based on genotypes from the 1000 Genome Project. The results suggest that such effects are non-existent or negligible (Table S11).

### Replication Rate

**Figure 4:**
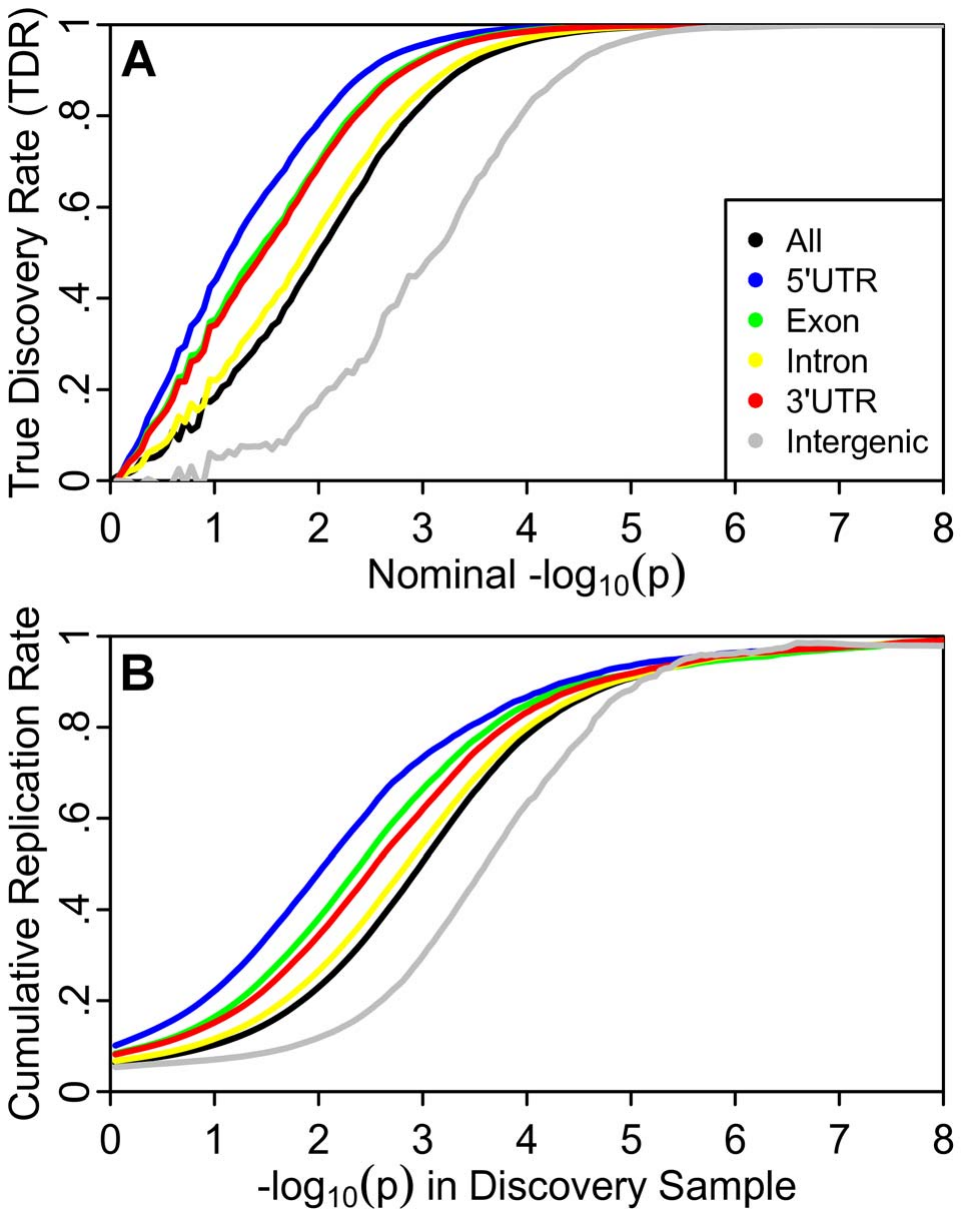
Independent study replication confirms enrichment in Crohn's disease. (A). Stratified True Discovery Rate (TDR) plots illustrating the increase in TDR associated with increased enrichment. (B) Cumulative replication plot showing the average rate of replication (p<.05) within sub-studies for a given p-value threshold shows enriched categories replicate at a higher rate in independent samples. The vertical intercept is the overall replication rate per category.

To further address the possibility that the observed pattern of differential enrichment results from spurious (i.e., non-generalizable) associations due to category-specific confounding effects or statistical modeling errors, we also studied the empirical replication rate across independent sub-studies for one phenotype (CD) where the required sub-study summary statistics were available. Figure 4A shows the estimated TDR curves for different annotation categories in CD, with a similar pattern as that described for in height, SCZ and CPD, above. TDR is an estimate of the expected replication rate for a sufficiently large replication sample. We hypothesized that strata with higher TDR for a given nominal p-value would also show higher empirical replication rate. Figure 4B shows the empirical cumulative replication rate plots as a function of nominal p-value for the same categories as for the stratified TDR plot in Figure 4A. Consistent with the category-specific TDR pattern, we found that the nominal p-value corresponding to a wide range of replication rates was 100 times higher for intergenic relative to the most enriched genic category (5′UTR). Similarly, SNPs from genic annotation categories showing the greatest enrichments replicated at higher rates, up to five times higher than intergenic for 5′UTR SNPs, independent of p-value thresholds. The increase in replication rate was found to be greatest for SNPs that do not meet genome-wide significance, suggesting that adjusting p-value thresholds according to the estimated category-specific TDR could greatly improve the discovery of replicating SNP associations.

### Increased Power Using Stratified False Discovery Rate

In order to demonstrate the utility of the enriched category information for improved discovery, we leveraged an established method for computing stratified False Discovery Rates [9]. The sFDR method improves the power of traditional methods for FDR control [22] by taking advantage of pre-defined, differentially enriched strata among multiple hypothesis testing p-values. Here, we define an increase in power from using stratified (vs. unstratified) methods as a decreased Non-Discovery Rate (NDR) for a given level of FDR control α, where NDR is the proportion of false negatives among all non-null tests [23]. Specifically, the ratio of 1-NDR from stratified FDR control to 1-NDR from unstratified FDR control captures the relative power of the two approaches. This ratio can be shown to be equivalent to the ratio of the number of SNPs rejected by sFDR to the number rejected by unstratified FDR for a common level α.

**Figure 5:**
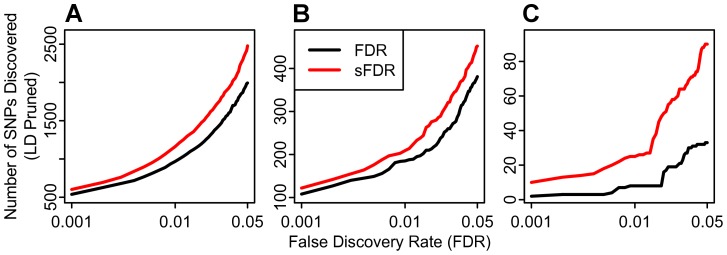
Enrichment improves discovery using established methods. Among three phenotypes, (A) Height, (B) Crohn's Disease, (C) and Schizophrenia, we demonstrate an increased discovery of SNPs at a given FDR when incorporating the enriched genic annotation information into an established stratified false discovery rate (sFDR; red) framework. SNPs declared significant by sFDR also replicate at a higher rate (Figure S12).

For each phenotype we divided the SNPs into independent strata according to its predicted tagged variance (z^2^). Tagged variance was predicted using on a linear model with regression weights for each annotation category trained using the height GWAS summary statistics. The enrichment of these strata is presented in Figure S11. In Figure 5 (and Table S12) we show an increase in the number of discovered SNPs. For example, for α = .05 the increased proportion of declared non-null SNPs using sFDR ranges from 20% in height to 300% in schizophrenia. Leveraging our genic annotation categories in the sFDR framework provides one possible avenue for improving the output of likely non-null SNPs in GWAS by taking advantage of the non-exchangeability of SNPs demonstrated by our enrichment analyses. Other formulations of strata and continued investigations into enrichment are likely to further improve the power of this approach.

## Discussion

Our results show a significant and consistent pattern of enrichment among genic elements, particularly the 5′UTR, exon and 3′UTR categories, for association with diverse complex traits and disorders. Intergenic SNPs were depleted more than tenfold. This has important analytical and conceptual implications. The results suggest that all tag SNPs should not be treated as exchangeable, but rather functional annotations of the underlying tagged SNPs can be leveraged in SNP discovery. Moreover, the results point to a common functional nature of the polygenetic architecture across diverse complex phenotypes.

GWAS have traditionally treated all SNPs as exchangeable, implicitly assigning all SNPs equal *a priori* probability of association. The current findings suggest that this assumption of exchangeability is not valid, and that the traditional statistical approaches to GWAS are highly suboptimal. Sun et al [13] have laid the groundwork for incorporating this non-exchangeability into Hypothesis Driven Genome-Wide Association Studies (HD-GWAS), and further applications and development of such methods is likely to prove fruitful. To illustrate the utility of these approaches we used our LD-weighted tag SNP annotations, combined with an established method for computing stratified False Discovery Rates, to demonstrate improved discovery of SNPs in GWAS under this testing framework. Annotation categories were chosen based on previous literature suggesting that SNPs in and near genes are likely to harbor many true polygenic effects. We provide a proof of principle, using intuitive categories, that *a priori* information about SNPs, irrespective of phenotype, can improve discovery of likely non-null SNPs. Given the wealth of information available for SNP variants, it is likely that other annotation schemes will potentially yield even greater enrichment and further increase the gains of our basic approach. We expect this approach will be of particular importance in polygenic complex phenotypes. Only a small fraction of the heritability is explained by currently discovered variants but converging evidence suggests much more remains buried in GWAS (i.e., traits with a large “missing heritability”) [4] for these traits.

Moreover, the non-exchangeability of SNPs based on LD-weighted genic categories has important implications for the generalizability of estimated SNP effect sizes. In particular, SNPs in highly enriched categories will have effect size estimates that replicate strongly in independent samples, whereas SNPs in impoverished categories will have effect sizes that replicate weakly in independent samples [17] (Figure S10). Identifying SNPs with generalizable effects is crucial to improving the predictive power of polygenic risk scores that combine SNP effects to predict variation in complex traits and diseases in new samples [24]. Properly assessing the generalization performance of SNP effect sizes will be of high importance for personalized medicine based on polygenic risk scores.

While knowing which SNP categories are enriched for true associations can guide gene discovery, knowing which SNPs are unlikely to have an effect is also important and can guide control of spurious inflation through improved genomic inflation correction [19], [20]. We show how the genic enrichment pattern can be used for genomic inflation control in GWAS. By estimating genomic control from intergenic tag SNPs, we can minimize the contamination of inflation estimates from true polygenic associations. While emerging studies have suggested that polygenic effects are detectable in GWAS data [5], [6], particularly in and around genes [6], [16], and the presence of these effects is consistent with a skewed distribution of p-values [17], [25] that resembles spurious inflation [19], differential confounds among our categories could persist. We provided a null GWAS (Table S11) and found no indications of spurious enrichment due to differential MAF and LD structure among our categories. Further, if our findings were due to spurious category-specific inflation, including differential population stratification, one would not expect a mirroring increase in replication across independent samples (Figure 4), except under the extreme condition where the population structure of the discovery sample was mirrored in the replication sample. In addition, the variable shape of the enrichment, deviating at different points along the expected line for different phenotypes, is inconsistent with spurious variance inflation. It is also of importance to note that the presence of spurious category-specific inflation would imply that any GWAS association within an enriched category (i.e., tagging an exonic or UTR SNP) should be considered *less* reliable. Our findings are consistent with the presence of true polygenic effects, however, we cannot entirely rule out contributions of potential confounding effects or alternate hypotheses. It is highly implausible, however, that they would explain away both the described enrichment and increased replication.

Conceptually, our results support findings that aspects of the genetic architecture are consistent across phenotypes [26], and previous suggestions from both model organisms [27] and humans [6], [16] that polygenic contributions are greater from variants in and around genes. Our findings agree with emerging trends in model organisms [26] and post-hoc GWAS analyses [28]–[30] suggesting that quantitative traits are affected by a large but quantifiable number of polymorphisms, inconsistent with ‘infinitesimal’ models [31], but notably polygenic. We also show evidence that tagged variance is proportional to genotypic variance (Figures S6 and S7), which supports the notion that common variation explains an important part of the variability in common diseases and traits [32].

Our findings also suggest that regulatory genic elements may be particularly enriched for polygenic effects. This is in line with the most strongly associated SNPs from GWAS, which mainly tag regulatory genic elements [3]. The 5′UTR, specifically, is important for the regulation of gene expression [33] making it a compelling candidate for playing a causal role in complex trait variation [1]. Also, 5′UTRs are less conserved evolutionarily than coding regions [34], despite their noted functionality, pointing to a potential source for regulatory variation thought to drive evolutionary differences among primates [35] and other species. We also found a stronger enrichment of SNPs tagging exons compared to introns. However, because we are considering tag SNPs we can only speculate about the functional consequences of the underlying causal variants.

Exome sequencing studies have identified causal variants for Mendelian disorders by leveraging hypotheses about the genetic architecture of these traits and thus focusing on protein changing variants [36]. While methods are continually improving for predicting the functional consequences of coding changes, predicting regulatory function has remained a challenge. Future target capture methods, deep sequencing efforts and custom SNP array designs, as well as functional prediction efforts in complex traits may improve power and utility by adding focus to regulatory elements, in particular 5′UTRs. As other potential annotation categories, such as transcription factor binding sites, methylation targets, conservation/selection, and gene expression patterns, become better characterized the current analyses could be extended to include these.

## Materials and Methods

### Genome-Wide Association Study (GWAS) Data

Fourteen phenotypes, body mass index (BMI) [37], height, waist to hip ratio [38] (WHR), Crohn's disease [39] (CD), ulcerative colitis [40] (UC), schizophrenia [41] (SCZ), bipolar disorder [42] (BD), smoking behavior as measured by cigarettes per day [43] (CPD), systolic and diastolic blood pressure [44] (SBP, DBP), and plasma lipids [45] (triglycerides, TG, total cholesterol, TC, high density lipoprotein, HDL, low density lipoprotein, LDL), were considered. Genome-wide association study (GWAS) results were obtained as summary statistics (p-values or z-scores) from public access websites (BMI, Height, WHR, TC, TG, HDL, LDL), published supplementary material (SBP, DBP), or through collaborations with investigators (CD, UC, SCZ, BD). For CD, pre-meta-analysis, sub-study specific p-values and effect sizes (z-scores) were obtained from the study principal investigators. In total these studies considered more than 1.3 million phenotypic observations, but considerable sample overlap makes the number of unique individuals much less. For details, see Text S1 and Table S1.

### GWAS Summary Statistics Processing

The summary statistics from the respective GWAS meta-analyses, derived according to best practices, were used as-is. No further processing was performed, with the exception of intergenic inflation control (described below). Results from SNPs with reference SNP (rs) numbers that did not map to our 1000 genomes project (1KGP) reference panel were excluded.

### Positional Annotation Categories

Bi-allelic SNP genotypes from the European reference sample provided by the November 2010 release of Phase 1 of the 1KGP were obtained in pre-processed form from http://www.sph.umich.edu/csg/abecasis/MACH/download/. Using Plink version 1.07 [46], [47] 1KGP SNPs with a minor allele frequency less than 1%, missing in more than 5% of individuals and/or violating Hardy-Weinberg equilibrium (p<1×10^−6^) were excluded from the reference panel. Individuals missing more than 10% of genotypes were excluded.

Each remaining 1KGP SNP was assigned a single, mutually exclusive genic annotation category based on its genomic position (hg19). Genic annotation categories were: 1) 10,000 to 1,001 base pairs upstream (10 k Up); 2) 1,000 to 1 base pair upstream (1 k Up); 3) 5′ untranslated region (5′UTR); 4) exon; 5) intron; 6) 3′ untranslated region (3′UTR); 7) 1 to 1,000 base pairs downstream (1 k Down); 8) 1,001 to 10,000 base pairs downstream (10 k Down), all with reference to protein coding genes only. Annotations were assigned based on the first gene transcript listed in the UCSC known genes database [48]. In total 9,078,405 1KGP SNPs were assigned positional categories. All positional categories were scored 0 or 1. For further details see Text S1.

### Linkage Disequilibrium (LD)-Weighted Scoring

For each GWAS tag SNP a pairwise correlation coefficient approximation to LD (r^2^) was calculated for all 1KGP SNPs within 1,000,000 base pairs (1 Mb) of the tag SNP using Plink version 1.07 [46], [47]. LD scores were thresholded providing continuous valued estimates from 0.2 to 1.0; r^2^ values <0.2 were set to 0 and each SNP was assigned an r^2^ value of 1.0 with itself. LD-weighted annotation scores were computed as the sum of r^2^ LD between the tag SNP and all 1KGP SNPs positioned in a particular category. Each tag SNP was assigned to every LD-weighted annotation category for which its annotation score was greater than or equal to 1.0. The resulting LD-weighted annotation categories were not mutually exclusive such that each GWAS tag SNP could be annotated with multiple categories. Summary statistics describing the distribution of scores in each category for the 2,558,411 SNPs, representing the union of all GWAS considered, are provided in Tables S2 and S3. Figure S1 provides a schematic of our scoring algorithm. All analyses were repeated using a second set of LD thresholding parameters and found to be robust (Text S1 and Figures S13, S14, S15, S16).

### Intergenic SNPs

Intergenic SNPs were determined after LD-weighted scoring and defined as having LD-weighted annotations scores for each of the eight categories equal to zero. In addition they were defined to not be in LD with any SNPs in the 1KGP reference panel located within 100,000 base pairs of a protein coding gene, within a noncoding RNA, within a transcription factor binding site nor within a microRNA binding site. SNPs labeled intergenic were defined to be a specific collection of non-genic SNPs chosen to not represent any functional elements within the genome (including through LD). Because of how they are defined these SNPs are hypothesized to represent a collection of null associations. Other non-genic categories (1 k up, 10 k up, 1 k down and 10 k down) were included in our analyses to ensure SNPs not too far away from genes, but not within protein coding genes, were represented by non-genic categories and enrichment due to these SNPs was not solely attributed to LD with genic categories.

### Stratified Q-Q Plots and Enrichment

Q-Q plots compare two probability distributions. For each phenotype, for all SNPs and for each categorical subset, −log_10_ nominal p-values were plotted against −log_10_ empirical p-values. Leftward deflections of the observed distribution from the projected null line reflect increased tail probabilities in the distribution of test statistics (z-scores) and consequently an over-abundance of low p-values compared to that expected by chance. We qualitatively refer to this deflection as “enrichment” (Figure 1 and Figure 2, Figure S3).

We estimated the significance of the annotation enrichment using two sample Kolmogorov-Smirnov (KS) Tests to compare the distribution of test statistics in each genic annotation category to the distribution of the intergenic category, for each phenotype. SNPs were pruned randomly to approximate independence (r^2^<0.2) ten times and Table S5 reports the p-value corresponding to the median KS statistics from the ten comparisons.

### Intergenic Inflation Control

The empirical null distribution in GWAS is affected by global variance inflation due to factors including population stratification and cryptic relatedness [20] and deflation due to over-correction of test statistics for polygenic traits by standard genomic control methods [19]. We applied a control method leveraging only intergenic SNPs that are likely depleted for true associations. All p-values were converted into z-scores and, for each phenotype, the genomic inflation factor [20], λ_GC_, was estimated for intergenic SNPs. All test statistics were divided by λ _GC_.

The inflation factor λ_GC_ was computed as the median z-score squared divided by the expected median of a chi-square distribution with one degree of freedom or all phenotypes except CPD, where the .95 quantile was used in place of the median. For correction statistics see Table S4.

### Quantification of Categorical Enrichment

For each phenotype, enrichment was measured as the mean(z-score^2^−1) for each category and normalized by the largest value per phenotype. The mean(z-score^2^−1) is a conservative estimate of the variance attributable to non-null SNPs, given a standard normal null distribution and a non-null distribution symmetric around zero (see Text S1).

### Q-Q Plots and False Discovery Rate (FDR)

Enrichment seen in the conditional Q-Q plots can be directly interpreted in terms of the FDR. Specifically, for a given p-value cutoff, the Bayes FDR [17] is defined as
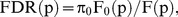
(1)where π_0_ is the proportion of null SNPs, F_0_ is the null cdf, and F is the cdf of all SNPs, both null and non-null. Under the null hypothesis, F_0_ is the cdf of the uniform distribution on the unit interval [0,1], so that Eq. [1] reduces to
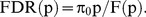
(2)The cdf F can be estimated by the empirical cdf q = N_p_/N/cdf F _p_ is the number of SNPs with p-values less than or equal to p, and N is the total number of SNPs. Replacing F by q and replacing π_0_ with unity in Eq. [2], we get
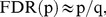
(3)This is upwardly biased, and hence p/q is conservative estimate of the FDR, and 1−p/q is a conservative estimate of the Bayes TDR [17]. If π_0_ is close to one, as is likely true for most GWAS, the increase in bias from setting π_0_ to one in Eq. [3] is minimal. Referring to the formulation of the Q-Q plots, we see that FDR(p) is equivalent to the nominal p-value under the null hypothesis divided by the empirical quantile of the p-values. Given the −log_10_ transformation applied to the Q-Q plots, we can easily read off
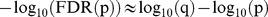
(4)demonstrating that the (conservatively) estimated FDR is directly related to the horizontal shift of the curves in the stratified Q-Q plots from the expected line x = y, with a larger shift corresponding to a smaller FDR. For the TDR plots in Figure 2, we estimated the TDR for each genic category according to Eq. [4].

Eq. [3] is the Empirical Bayes point estimate of the Bayes FDR given in Efron (2010). Using Eq. [3] to control FDR (i.e., the expected proportion of falsely rejected null hypotheses) [22] is closely related to the “fixed rejection region” approach of Storey [49], [50]. Specifically, Storey [49] showed, for a given FDR α, rejecting all null hypotheses such that p/q<α is equivalent to the Benjamini-Hochberg procedure and provides asymptotic control of the FDR to α if the true null p-values are independent and uniformly distributed. Storey [49] also noted that asymptotic control is preserved under positive blockwise dependence, whereas Schwartzman and Lin [51] showed that Eq. [3] is a consistent estimator of FDR for asymptotically sparse dependence (i.e. the proportion of correlated pairs of p-values goes to zero as the number of hypothesis tests becomes large). Sparse dependence is a good description of the dependence present in GWAS data; for example, based on a threshold of r^2^>.05 within 1,000,000 basepairs, we estimate the ratio of correlated pairs (r^2^>.05) to total pairs of p-values at 0.000128.

### Replication Rate

For each of eight sub-studies contributing to the final meta-analysis in the CD report we independently adjusted z-scores using intergenic inflation control. For each of 70 (8 choose 4) possible combinations of four-study discovery and four-study replication sets, we calculated the four-study combined discovery z-score and four-study combined replication z-score for each SNP as the average z-score across the four studies, multiplied by two (the square root of the number of studies). For discovery samples the z-scores were converted to two-tailed p-values, while replication samples were converted to one-tailed p-values preserving the direction of effect in the discovery sample. For each of the 70 discovery-replication pairs cumulative rates of replication were calculated over 1000 equally-spaced bins spanning the range of negative log_10_(p-values) observed in the discovery samples. The cumulative replication rate for any bin was calculated as the proportion of SNPs with a −log_10_(discovery p-value) greater than the lower bound of the bin with a replication p-value<.05. Cumulative replication rates were calculated independently for each of the eight genic annotation categories as well as intergenic SNPs and all SNPs. For each category, the cumulative replication rate for each bin was averaged across the 70 discovery-replication pairs and the results are reported in Figure 4. The vertical intercept is the overall replication rate.

### Stratified False Discovery Rates

A multiple linear regression was used to predict the tagged variance (z^2^) for each SNP in the height GWAS from the unthresholded LD-weighted annotation scores. Using the category weights determined from this ‘training’ regression on the height GWAS, the tagged variance for each SNP was predicted from its annotation vector for each other phenotype. For each phenotype, SNPs were grouped into strata according to the rank of this predicted tagged variance. Enrichment for each stratum was demonstrated using Q-Q plots as described above (Figure S11). We note that for Figure 5A the height data serves as both our ‘test’ (for creating strata) and ‘training’ (for detecting enrichment) data, but for each other GWAS the training and test data is independent. Sun et al [9] described a stratified False Discovery Rate (sFDR) procedure which can result in improved statistical power over traditional FDR methods [22] when a collection of statistical tests can be grouped into disjoint strata with different levels of enrichment. In order to demonstrate the utility of using genic annotation categories in combination with sFDR for increasing power, we computed the number of SNPs deemed significant at a given FDR threshold using both traditional [22] and stratified FDR, where the strata were determined by the predicted tagged variance for each SNP based on regression weights determined from the height GWAS summary statistics (Figure 5). The increase in rejections for a common threshold α when using sFDR is equivalent to increased power demonstrated by a ratio of one minus Non-Discovery Rates (1-NDRs) for sFDR to FDR greater than 1 [23].

We also computed the average proportion of SNPs above a given rank (i.e. top 1000) that replicated based on unadjusted and strata adjusted ranks (determined from the sFDR procedure) across the 70 permutations of four study discovery and four study replication samples possible in the eight study CD meta-analysis GWAS (Figure S12). These results demonstrate that for a given threshold, SNPs ranked via genic category-informed sFDR replicate in higher numbers than SNPs ranked via traditional FDR.

## Supporting Information

Figure S1 Annotation Category Scoring Schematic. For each GWAS tag SNP in the 1KGP we estimated r^2^ LD with all SNPs within 1 megabase. LD scores were thresholded at r^2^>0.2. For each SNP the sum of LD with each genic annotation category was recorded. SNPs were assigned to categories by thresholding continuous scores with an inclusive lower bound of 1.0. Positional (non LD-weighted) scores were recorded as the annotation for the GWAS tag SNP's location only.
(TIF)

Figure S2 Correlations among annotation categories and scores. (A) Heat map displaying the Spearman's correlation coefficients among continuous valued LD-weighted annotation scores. (B) Heat map displaying the Spearman's correlation coefficients among thresholded and binarized annotation categories presented in Q-Q plots. Correlations are reported using the annotations for the union of SNPs across all GWAS (2,558,411 SNPs).
(TIF)

Figure S3 Enrichment in Height without LD weighted annotation. Q-Q plot showing enrichment of genic annotation categories using positional scores (non LD-weighted). Enrichment patterns are present, but less apparent than using LD-weighted annotation scores (Figure 1). No inflation correction was performed by our group.
(TIF)

Figure S4 Height before and after Intergenic Inflation Control. (A) Q-Q plot of height without correction for genomic inflation. (B) Q-Q plot of height after correction for genomic inflation using the ‘intergenic inflation control’. Note the overcorrection (grey line below null-hypothesis line, marked by red arrows) in the un-corrected Q-Q plot in Panel A is resolved in Panel B. Although slight, because of the log scaling of these plots, this slight deflation (left of 1.5 on the x-axis of Panel A) occurs over a much greater proportion of the distribution and thus has a stronger effect on the mean and median of the distribution than the more visually apparent inflation in the extreme tails (right of 2 on the x-axis of Panel A). For lambda values, see Table S4. Only nominal p-values below the standard genome-wide significance threshold (p<5×10^−8^) are shown.
(TIF)

Figure S5 Categorical enrichment for all phenotypes. The mean(z-score^2^−1) for each category of SNPs per phenotype reveals consistent enrichment across fourteen phenotypes. BD, Bipolar Disorder; BMI, Body Mass Index; CD, Crohn's disease; CPD, Cigarettes per Day; DBP, Diastolic blood pressure; HDL, High density lipoprotein; LDL, Low density lipoprotein; SBP, systolic blood pressure; SCZ, Schizophrenia; TC, total Cholesterol; TG, triglycerides; UC, Ulcerative Colitis; WHR, Waist-hip-ratio.
(TIF)

Figure S6 Mean Z^2^ per decile of genetic variance by category. The figure shows the relationship between genetic variance defined as a function of minor allele frequnecy (MAF) by MAF×(1-MAF) and effect size (mean z-score^2^) per genic annotation category for height. The mean is taken at each decile of genetic variance. The red lines are fits from generalized additive models to the mean mean observed squared z-scores. Note the clear increase in effect size with increasing MAF, which shows similar effect of MAF across categories. The exception is for the intergenic category which shows little multiplicative effect further suggesting it harbors a majority of true null SNPs.
(TIF)

Figure S7 Enrichment by MAF category for height and schizophrenia. The effect of minor allele frequency (MAF) is not consistent across phenotypes. (A) For height more common SNPs show a continually larger enrichment than less common SNPs. (B) For Schizophrenia common SNPs show more enrichment at moderate to small z-scores, but for larger z-scores less common SNPs are more enriched.
(TIF)

Figure S8 locfdr plot for all SNPs in Crohn's disease. Mixture model fits for all SNPs for Crohn's disease. Black: empirical z-score distribution, Purple: estimated non-null distribution, Green line: smooth estimate of mixture distribution (full distribution), Blue line: smooth estimate of the null distribution. Diamonds: local false discovery rate (LFDR) of 0.2. The estimated proportion of non-null SNPs varies by category, as does the variance in the estimated non-null effect size.
(TIF)

Figure S9 locfdr plots for genic annotation categories in Crohn's disease. Mixture model fits for each annotation category for Crohn's disease. Black: empirical z-score distribution, Purple: estimated non-null distribution, Green line: smooth estimate of mixture distribution (full distribution), Blue line: smooth estimate of the null distribution. Diamonds: local false discovery rate (LFDR) of 0.2. The estimated proportion of non-null SNPs varies by category, as does the variance in the estimated non-null effect size.
(TIF)

Figure S10 Z-score–z-score plots confirm mixture model predictions. (A) Expected *a posteriori* estimates of effect size for a given observed z-score. (B) Z-score-z-score plot demonstrates the empirical replication z-scores closely match the expected *a posteriori* effect sizes and are strongly dependent upon genic annotation category.
(TIF)

Figure S11 Regression based strata enrichment. Q-Q plot enrichment for the regression based strata for (A) Height, (B) Crohn's Disease (CD), and (C) Schizophrenia (SCZ). SNPs predicted to have a higher tagged variance (z^2^) show greater levels of enrichment. Enrichment is consistent across these three phenotypes, despite being determined from a regression model only using the summary statistics from the height GWAS.
(TIF)

Figure S12 Replication Rates of sFDR versus FDR. For a given SNP rank threshold (i.e., top 500 SNPs), those ranked by the genic annotation category-informed stratified FDR show a greater absolute number of replications, and thus a greater rate of replication, when compared to the annotation un-informed standard FDR. When incorporated into an FDR framework, the enriched genic annotation categories lead to an increased rate of true discovery among SNPs at a given cut off.
(TIF)

Figure S13 Stratified QQ-plots with different scoring parameters. The original stratified QQ-plots for height (A), Schizophrenia (B), and Cigarettes per day (C) using LD-weighted annotation categories created from an LD matrix describing the pairwise correlation between each GWAS SNP and all 1000 genomes SNPs (described above) including r^2^ values greater than 0.2 and within 1 megabase of the target GWAS SNP show a qualitatively similar pattern of enrichment when the scoring parameters are changed to include all pairwise r^2^ values greater than 0.05 and within 2 megabases (Height, D; Schizophrenia, E; Cigarettes per day, F).
(TIF)

Figure S14 Mean(z-score^2^−1) plot with alternate scoring parameters. The patterns among the mean(z-score^2^−1) for each category of SNPs per phenotype is robust to LD-weighted annotation scoring parameters as the patterns match those shown in Figure S6. Here we show results when pairwise LD is thresholded at r^2^>0.05 and within 2 megabases (original scoring: r^2^>0.2 and within 1 megabase). BD, Bipolar Disorder; BMI, Body Mass Index; CD, Crohn's disease; CPD, Cigarettes per Day; DBP, Diastolic blood pressure; HDL, High density lipoprotein; LDL, Low density lipoprotein; SBP, systolic blood pressure; SCZ, Schizophrenia; TC, total Cholesterol; TG, triglycerides; UC, Ulcerative Colitis; WHR, Waist-hip-ratio.
(TIF)

Figure S15 Replication rate among categories with alternate scoring parameters. A regenerated cumulative replication plot (Figure 4B) showing the average rate of replication (p<.05) within independent sub-studies for a given p-value. The enrichment produced by the alternate LD weighted annotation scoring parameters (including r^2^>0.05 and all SNPs within 2 megabases) results in a similar pattern of increased replication as with the original parameters (including r^2^>0.2 and all SNPs within 1 megabases), with the exception of the intergenic category, which shows a noticeable decrease in the replication rate.
(TIF)

Figure S16 Relationship between total categorical total LD and z-score^2^. The mean (z^2^) of each category, using the height GWAS, as we change the threshold for inclusion for both the original (A; including r^2^>0.2 and within 1 megabases), and alternate (B; r^2^>0.05 and within 2 megabases) parameters for LD weighted scoring. The mean(z^2^) increases approximately monotonically each category, but with noticeably different slopes. The 5′UTR category in figure A becomes unstable at high thresholds because there are very few SNPs remaining. Changing to a more inclusive LD weighted scoring increases the number of SNPs with high scores and improves the relationship. This suggests that even greater enrichment could be achieved by tuning the categorical inclusion threshold upwards.
(TIF)

Figure S17 Parametric mixture model fits to Q-Q plots. Q-Q Plot for Height (A) and Crohn's Disease (B). Solid black lines are actual data. Dotted black lines are Q-Q curves under the global null hypothesis. Solid red lines are fitted Q-Q curves from Weibull mixture model for transformed p-values. Note, upper limit in Q-Q plot y-axes is 7.3, corresponding to GWAS-significance threshold of p = 5×10^−8^.
(TIF)

Figure S18 Effect of non-null proportion on Q-Q plots. Predicted Q-Q Plot for Crohn's Disease (CD; solid black line) from parametric Weibull mixture model fit (model given by Equation [S9]). The blue line is the predicted Q-Q curve of the CD data if the non-null proportion π_1_ were 0.001 instead of the value 0.026 estimated from the CD data. The red line is the predicted Q-Q curve if the non-null proportion π_1_ were 0.10.
(TIF)

Figure S19 Effect of sample size on Q-Q plots. Predicted Q-Q Plot for Crohn's Disease (CD; solid black line) from parametric Weibull mixture model fit (model given by Equation [S9]). The blue line is the predicted Q-Q curve of the CD data if the sample size were half as large as the true sample size (n = 51,109). The red line is the predicted Q-Q curve of the CD data if the sample size were five times as large as the true sample size.
(TIF)

Table S1 Descriptive statistics for each GWAS study. All traits are highly heritable and summary statistics are from well-powered studies. All Studies were imputed with using the HapMap phase II as a reference, with the exception of CD, UC and SCZ that used HapMap phase III as a reference. These statistics describe the results of the study in the form they were obtained by our group.
(XLSX)

Table S2 LD-weighted score distribution for the union of SNPs across all studies. The average score for different categories varies widely and reflects the relative abundance of the different elements within the genome. *Note intergenic scores are binary, with a score of 1 denoting an intergenic SNP.
(XLSX)

Table S3 The number of SNPs per annotation category. The table shows the number of tag SNPs in each annotation category from each GWAS without LD based annotation (using only positional information (No LD) and after LD based annotation (LD). Note the increased number of SNPs in all annotation categories, especially in annotation categories such as 3′UTR and 5′UTR when using LD-weighted categories. BD, Bipolar Disorder; BMI, Body Mass Index; CD, Crohn's disease; CPD, Cigarettes per Day; DBP, Diastolic blood pressure; HDL, High density lipoprotein; LDL, Low density lipoprotein; SBP, systolic blood pressure; SCZ, Schizophrenia; TC, total Cholesterol; TG, triglycerides; UC, Ulcerative Colitis; WHR, Waist-hip-ratio.
(XLSX)

Table S4 Estimated genomic inflation factors before and after intergenic inflation control (IIC). We present the estimates from either all SNPs or intergenic SNPs. The λ_GC_ values calculated before IIC were calculated from the summary statistics as they were made available to us, either by collaborators or public data repositories. Many of these studies already had performed a standard genomic control procedure, adjusting the test statistics down, to correct for inflation. For these studies our procedure may correct statistics upwards, increasing the computed λ_GC_ values. We leveraged the intergenic SNPs to estimate inflation because their relative depletion of associations suggests they provide a robust estimate of true null SNPs that is less contaminated by polygenic effects. Using annotation categories in this fashion is important given concerns posed by recent GWAS [8] about the over-correction of test statistics using standard genomic control [15]. Values greater than 1 indicate inflation and values less than 1 indicate an over correction, relative to the theoretical empirical null distribution. λ_GC_ was calculated as the ratio of the median z-score^2^ to the expected median of a Chi-square distribution with 1 degree of freedom, for all SNPs and intergenic SNPs independently. IIC, Intergenic Inflation Control; BD, Bipolar Disorder; BMI, Body Mass Index; CD, Crohn's disease; CPD, Cigarettes per Day; DBP, Diastolic blood pressure; HDL, High density lipoprotein; LDL, Low density lipoprotein; SBP, systolic blood pressure; SCZ, Schizophrenia; TC, total Cholesterol; TG, triglycerides; UC, Ulcerative Colitis; WHR, Waist-hip-ratio.
(XLSX)

Table S5 Significance of QQ-plot enrichment. The p-values of the enrichment of the Q-Q plots for all phenotypes compare intergenic annotation category with each other annotation category. Each p-value corresponds to the median Kolmogorov-Smirnov (KS) statistic from 10 iterations of each comparison for 10 different random prunings of SNPs to approximate independence (r^2^<0.2). We note that the KS test used is known to be overly conservative when distributions differ only in the extreme tails. The results from all categories for CPD, although consistent in direction with each other phenotype, do not reach significance likely because the differences are most strongly confined to the extreme tails. BD, Bipolar Disorder; BMI, Body Mass Index; CD, Crohn's disease; CPD, Cigarettes per Day; DBP, Diastolic blood pressure; HDL, High density lipoprotein; LDL, Low density lipoprotein; SBP, systolic blood pressure; SCZ, Schizophrenia; TC, total Cholesterol; TG, triglycerides; UC, Ulcerative Colitis; WHR, Waist-hip-ratio.
(XLSX)

Table S6 Enrichment Scores. Here we present in a table the enrichment values used to create Figure 2 and Figure S6, the normalize mean(z-score^2^–1). All values are expressed in relative proportions of the highest category for each phenotype. BD, Bipolar Disorder; BMI, Body Mass Index; CD, Crohn's disease; CPD, Cigarettes per Day; DBP, Diastolic blood pressure; HDL, High density lipoprotein; LDL, Low density lipoprotein; SBP, systolic blood pressure; SCZ, Schizophrenia; TC, total Cholesterol; TG, triglycerides; UC, Ulcerative Colitis; WHR, Waist-hip-ratio.
(XLSX)

Table S7 Per category average per SNP total tagged LD. The average total LD score for GWAS tag SNPs per LD-weighted genic annotation category for each phenotype is shown. Total LD is measured as the sum of pairwise LD scores (r^2^>.2) relating each GWAS tag SNP to all 1KGP SNPs within 1,000,000 base pairs. Note the consistent pattern across phenotypes, with large variation between annotaion categories, with highest LD score in 5′UTR. BD, Bipolar Disorder; BMI, Body Mass Index; CD, Crohn's disease; CPD, Cigarettes per Day; DBP, Diastolic blood pressure; HDL, High density lipoprotein; LDL, Low density lipoprotein; SBP, systolic blood pressure; SCZ, Schizophrenia; TC, total Cholesterol; TG, triglycerides; UC, Ulcerative Colitis; WHR, Waist-hip-ratio.
(XLSX)

Table S8 Per category average per SNP number of tagged SNPs. The average total number of SNP tagged (r^2^>0.2) by a tag SNP per genic annotation category for each phenotype is shown. Note the consistent pattern across phenotypes, with variation between categories, and highest number in 5′UTR. The distribution of block sizes does match the ordering of enrichment by category. BD, Bipolar Disorder; BMI, Body Mass Index; CD, Crohn's disease; CPD, Cigarettes per Day; DBP, Diastolic blood pressure; HDL, High density lipoprotein; LDL, Low density lipoprotein; SBP, systolic blood pressure; SCZ, Schizophrenia; TC, total Cholesterol; TG, triglycerides; UC, Ulcerative Colitis; WHR, Waist-hip-ratio.
(XLSX)

Table S9 Per category average MAF. The average minor allele frequency of GWAS tag SNPs in each genic annotation category for every phenotype is not consistent with this effect driving our enrichment patterns. Note the similarities across phenotypes and annotation categories. BD, Bipolar Disorder; BMI, Body Mass Index; CD, Crohn's disease; CPD, Cigarettes per Day; DBP, Diastolic blood pressure; HDL, High density lipoprotein; LDL, Low density lipoprotein; SBP, systolic blood pressure; SCZ, Schizophrenia; TC, total Cholesterol; TG, triglycerides; UC, Ulcerative Colitis; WHR, Waist-hip-ratio.
(XLSX)

Table S10 Multiple regression analysis predicting log(Z^2^) in height. A multiple regression analysis reveals a minimal, but significant, effect of total LD on the log z^2^ for height. This represents a minimal, but significant, effect of overall LD block size on enrichment. Categorical effects remain independently strong in this analysis with an effect size order that mirrors enrichment.
(DOCX)

Table S11 Null GWAS Simulations. We present simulations of categorical enrichment based on multiple independent null GWAS simulations using subjects with European ancestry from the 1000 Genomes Project. Random phenotypes were generated unrelated to genotypes for each subject, association z-scores were computed for each tag SNP, and mean(z^2^) was computed for each annotation category, using the same procedure as applied to the actual GWAS data. The means and standard deviations were computed from 20 independent simulation runs. The results demonstrate that the observed differential enrichment of annotation categories cannot be explained by category-specific spurious sources of genomic inflation due to differential LD or MAF.
(DOCX)

Table S12 FDR versus sFDR Discovery. Leveraging the enriched genic annotation categories to create strata among the SNPs we show that the stratified false discovery rate (sFDR) method improves the discovery of SNPs for a given FDR threshold, across all phenotypes. The numbers reported are after pruning SNPs for LD at a threshold of r^2^≤0.2.
(XLSX)

Text S1 Supplementary text extending Materials and Methods and presenting supporting analyses. More details are provided with respect to the acquisition, processing and annotating of the GWAS data used for the main results. The relationship between QQ-plots and False Discovery Rate is extended and related to our measures of enrichment. Also, the main results are described within the context of a mixture-modeling framework. Finally, a series of control experiments are described and supplementary references are enumerated.
(DOCX)
