# Supplementary figures and images for: All SNPs Are Not Created Equal: Genome-Wide Association Studies Reveal a Consistent Pattern of Enrichment among Functionally Annotated SNPs
Source: PLoS Genet. 2013 Apr 25;9(4):e1003449. doi: 10.1371/journal.pgen.1003449 (PMC3636284; doi:10.1371/journal.pgen.1003449)

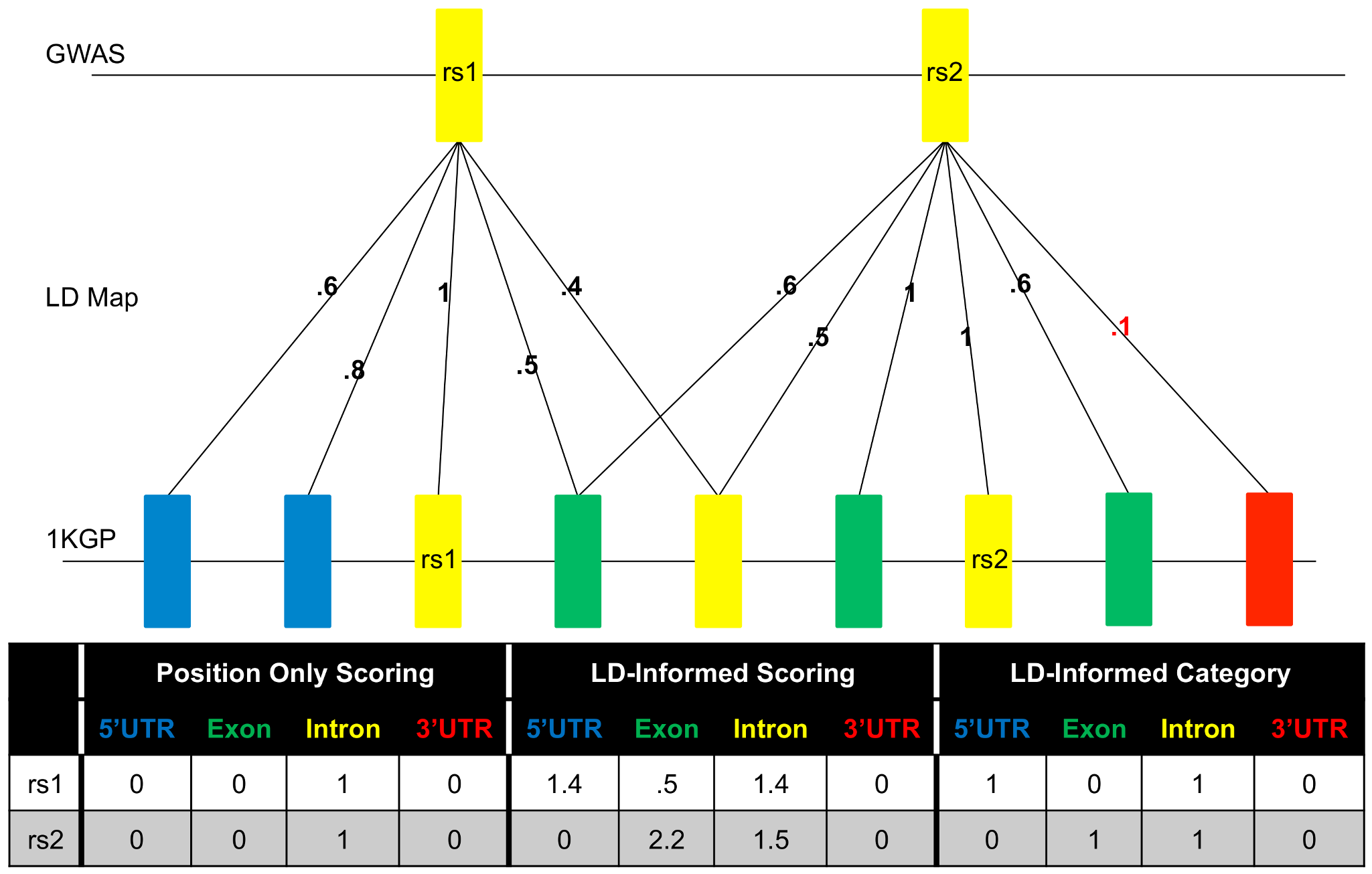

Supplement: Figure S1 — Annotation Category Scoring Schematic. For each GWAS tag SNP in the 1KGP we estimated r2 LD with all SNPs within 1 megabase. LD scores were thresholded at r2>0.2. For each SNP the sum of LD with each genic annotation category was recorded. SNPs were assigned to categories by thresholding continuous scores with an inclusive lower bound of 1.0. Positional (non LD-weighted) scores were recorded as the annotation for the GWAS tag SNP's location only. (TIF) [file pgen.1003449.s001.tif]

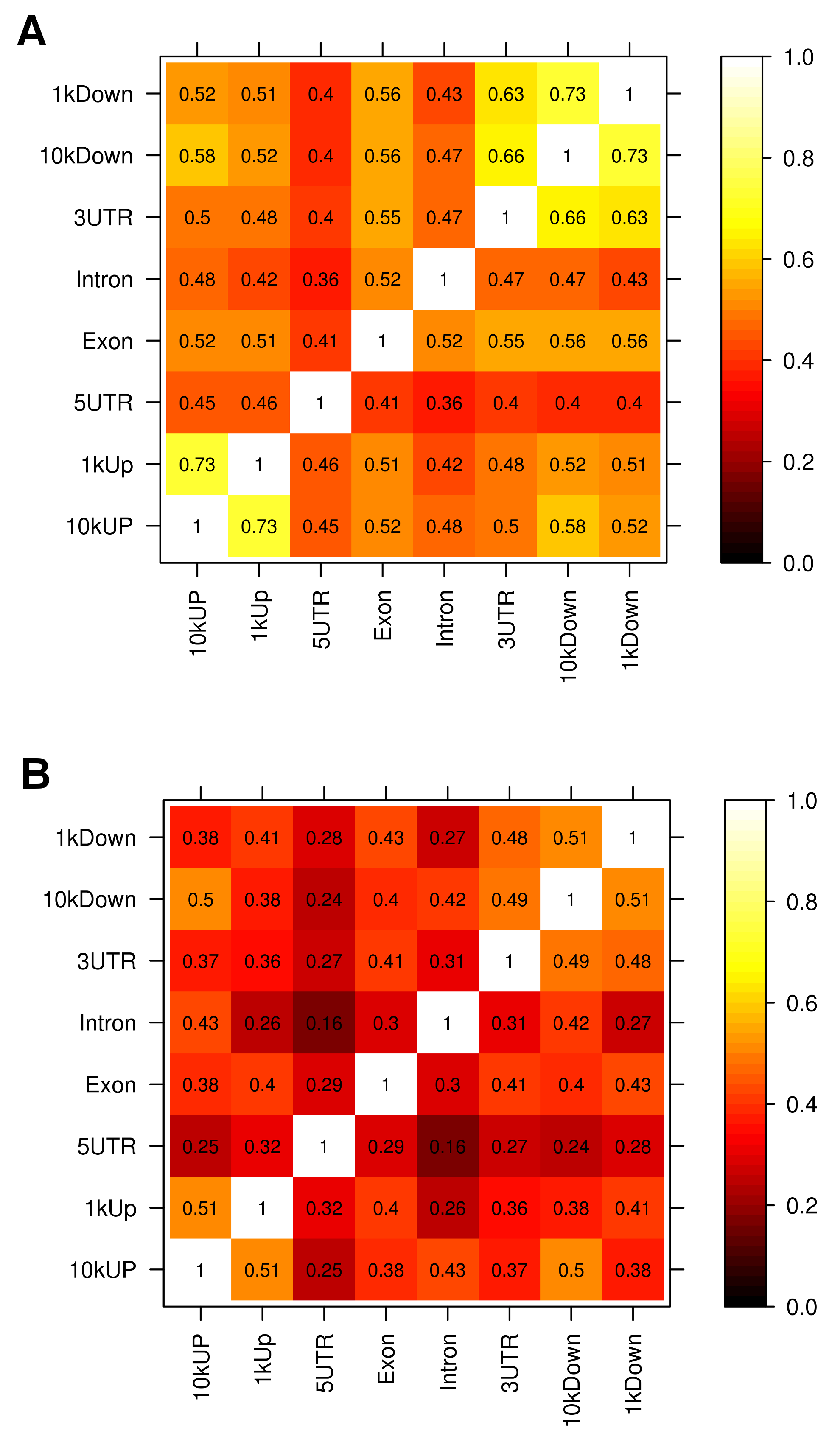

Supplement: Figure S2 — Correlations among annotation categories and scores. (A) Heat map displaying the Spearman's correlation coefficients among continuous valued LD-weighted annotation scores. (B) Heat map displaying the Spearman's correlation coefficients among thresholded and binarized annotation categories presented in Q-Q plots. Correlations are reported using the annotations for the union of SNPs across all GWAS (2,558,411 SNPs). (TIF) [file pgen.1003449.s002.tif]

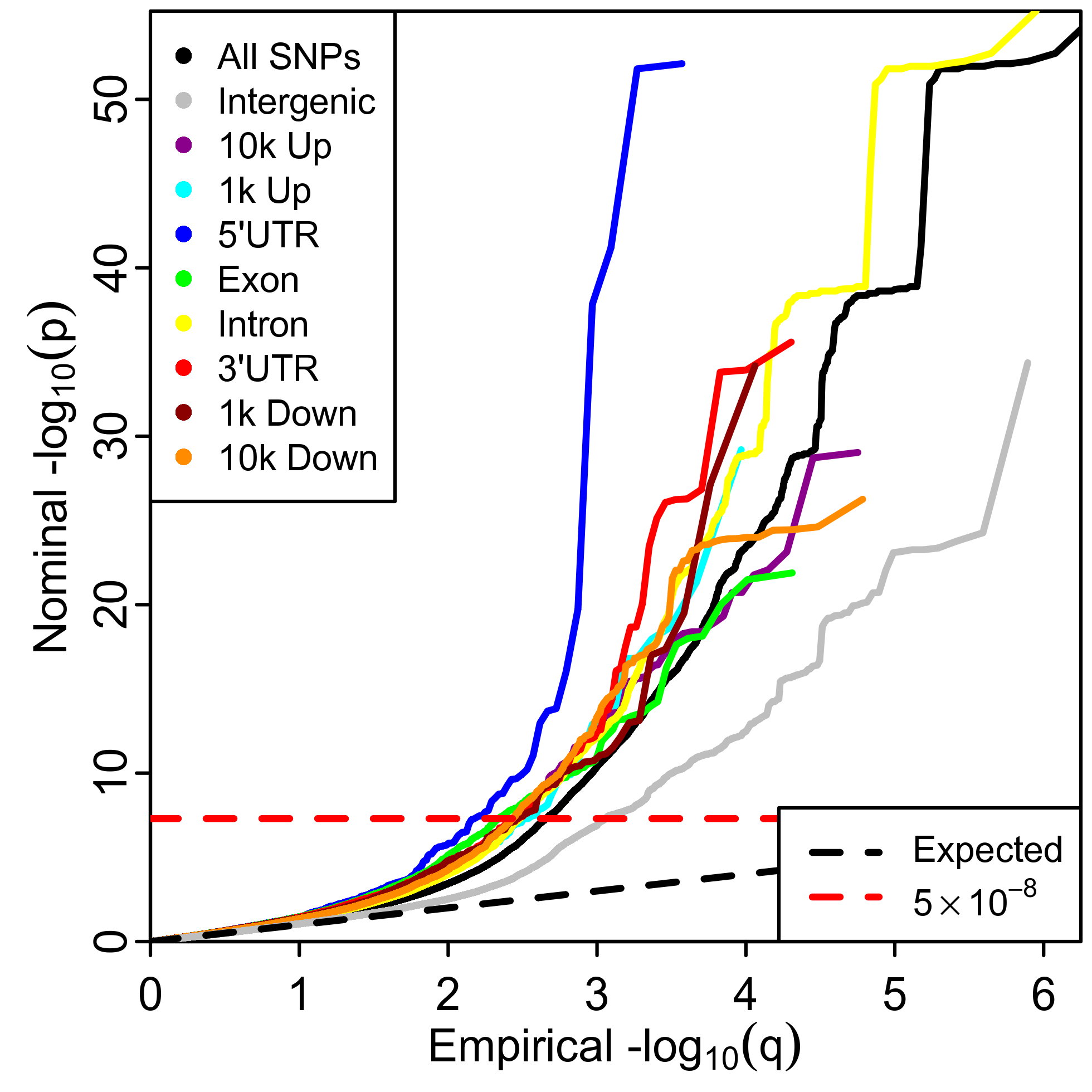

Supplement: Figure S3 — Enrichment in Height without LD weighted annotation. Q-Q plot showing enrichment of genic annotation categories using positional scores (non LD-weighted). Enrichment patterns are present, but less apparent than using LD-weighted annotation scores (Figure 1). No inflation correction was performed by our group. (TIF) [file pgen.1003449.s003.tif]

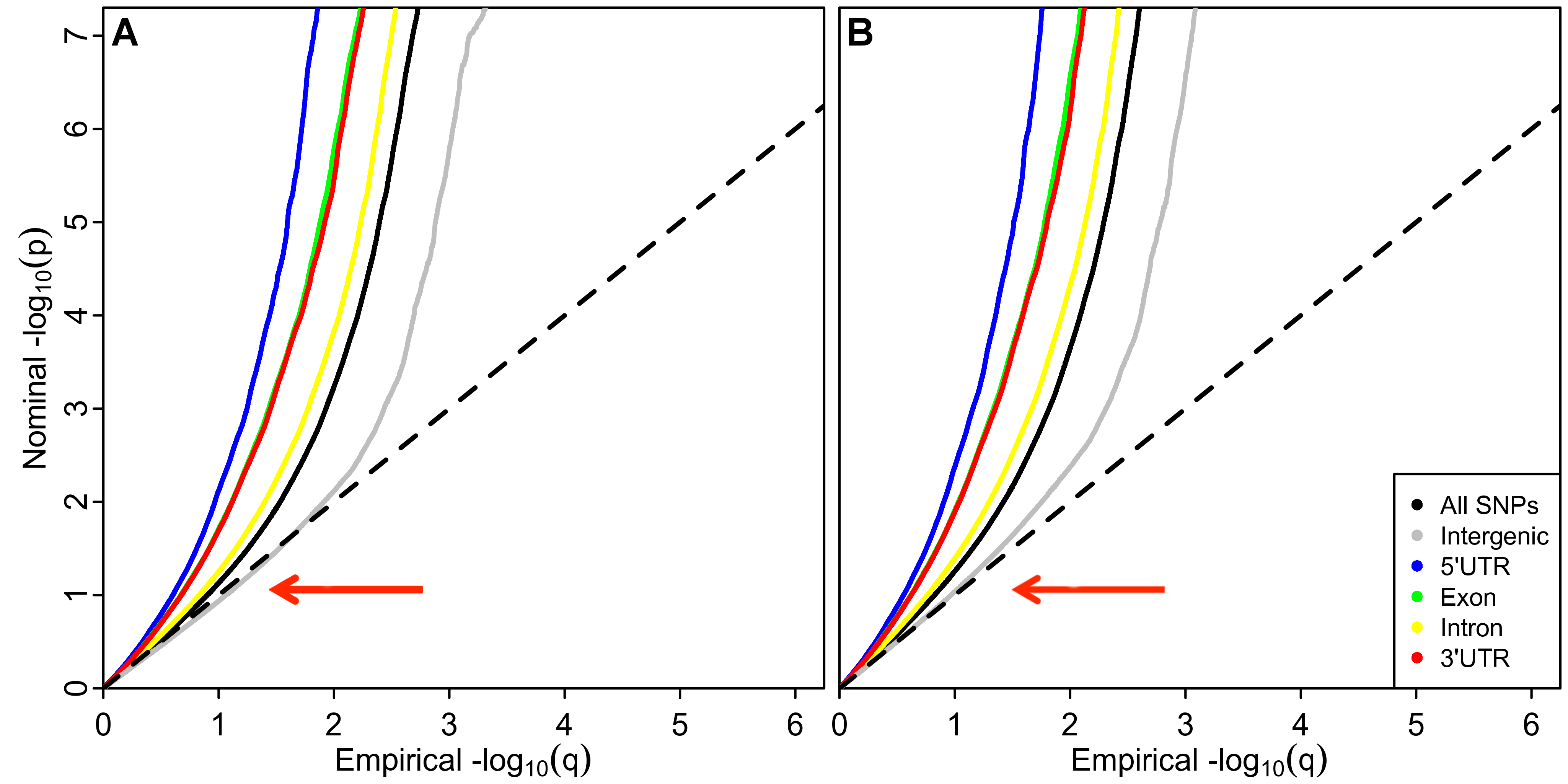

Supplement: Figure S4 — Height before and after Intergenic Inflation Control. (A) Q-Q plot of height without correction for genomic inflation. (B) Q-Q plot of height after correction for genomic inflation using the ‘intergenic inflation control’. Note the overcorrection (grey line below null-hypothesis line, marked by red arrows) in the un-corrected Q-Q plot in Panel A is resolved in Panel B. Although slight, because of the log scaling of these plots, this slight deflation (left of 1.5 on the x-axis of Panel A) occurs over a much greater proportion of the distribution and thus has a stronger effect on the mean and median of the distribution than the more visually apparent inflation in the extreme tails (right of 2 on the x-axis of Panel A). For lambda values, see Table S4. Only nominal p-values below the standard genome-wide significance threshold (p<5×10^−8^) are shown. (TIF) [file pgen.1003449.s004.tif]

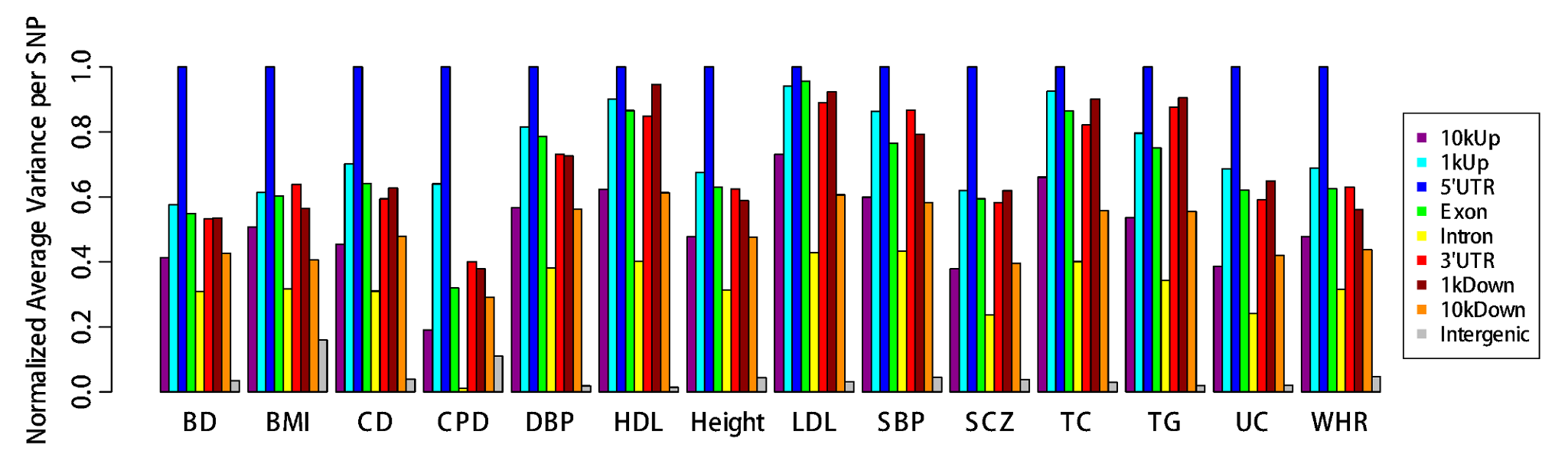

Supplement: Figure S5 — Categorical enrichment for all phenotypes. The mean(z-score2−1) for each category of SNPs per phenotype reveals consistent enrichment across fourteen phenotypes. BD, Bipolar Disorder; BMI, Body Mass Index; CD, Crohn's disease; CPD, Cigarettes per Day; DBP, Diastolic blood pressure; HDL, High density lipoprotein; LDL, Low density lipoprotein; SBP, systolic blood pressure; SCZ, Schizophrenia; TC, total Cholesterol; TG, triglycerides; UC, Ulcerative Colitis; WHR, Waist-hip-ratio. (TIF) [file pgen.1003449.s005.tif]

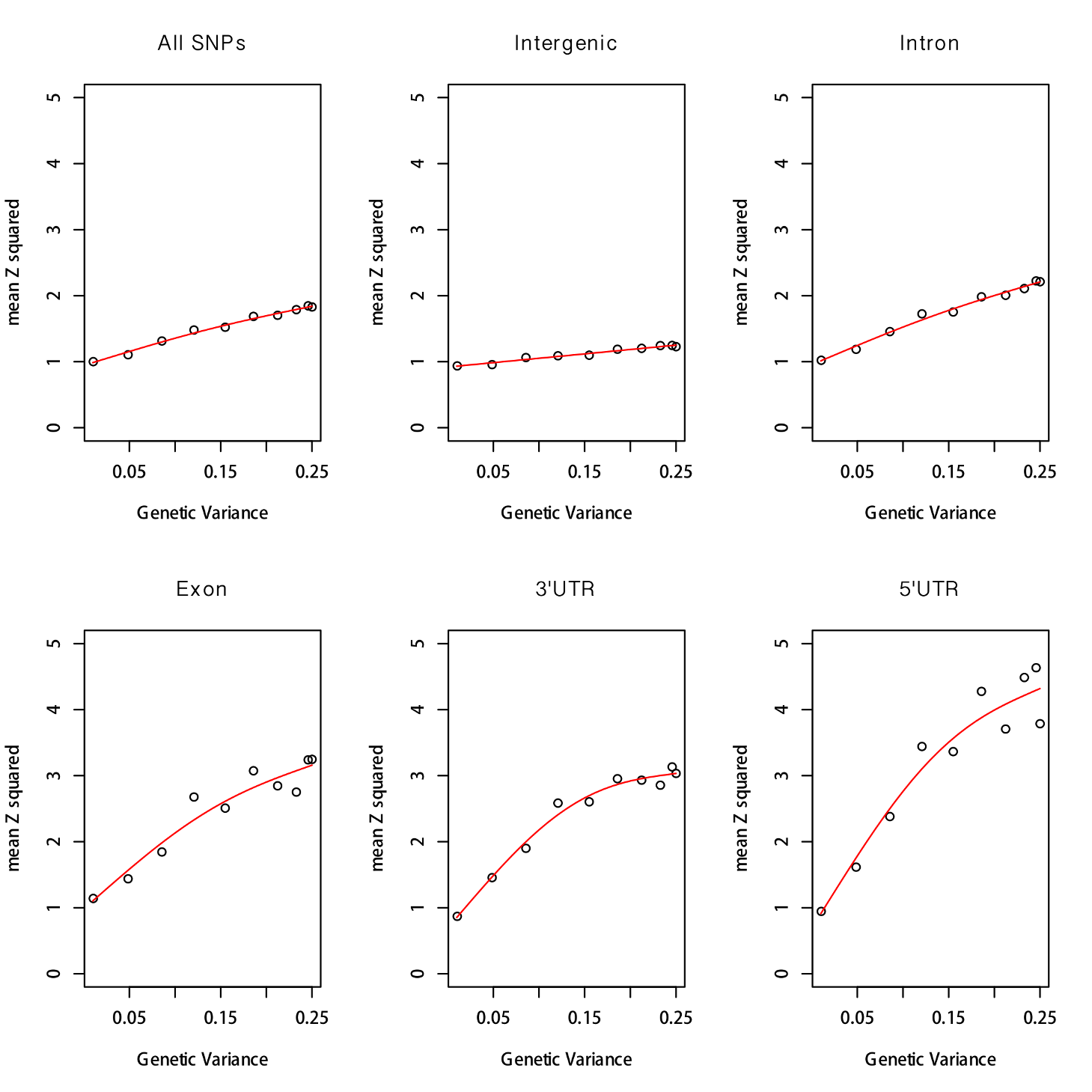

Supplement: Figure S6 — Mean Z2 per decile of genetic variance by category. The figure shows the relationship between genetic variance defined as a function of minor allele frequnecy (MAF) by MAF×(1-MAF) and effect size (mean z-score2) per genic annotation category for height. The mean is taken at each decile of genetic variance. The red lines are fits from generalized additive models to the mean mean observed squared z-scores. Note the clear increase in effect size with increasing MAF, which shows similar effect of MAF across categories. The exception is for the intergenic category which shows little multiplicative effect further suggesting it harbors a majority of true null SNPs. (TIF) [file pgen.1003449.s006.tif]

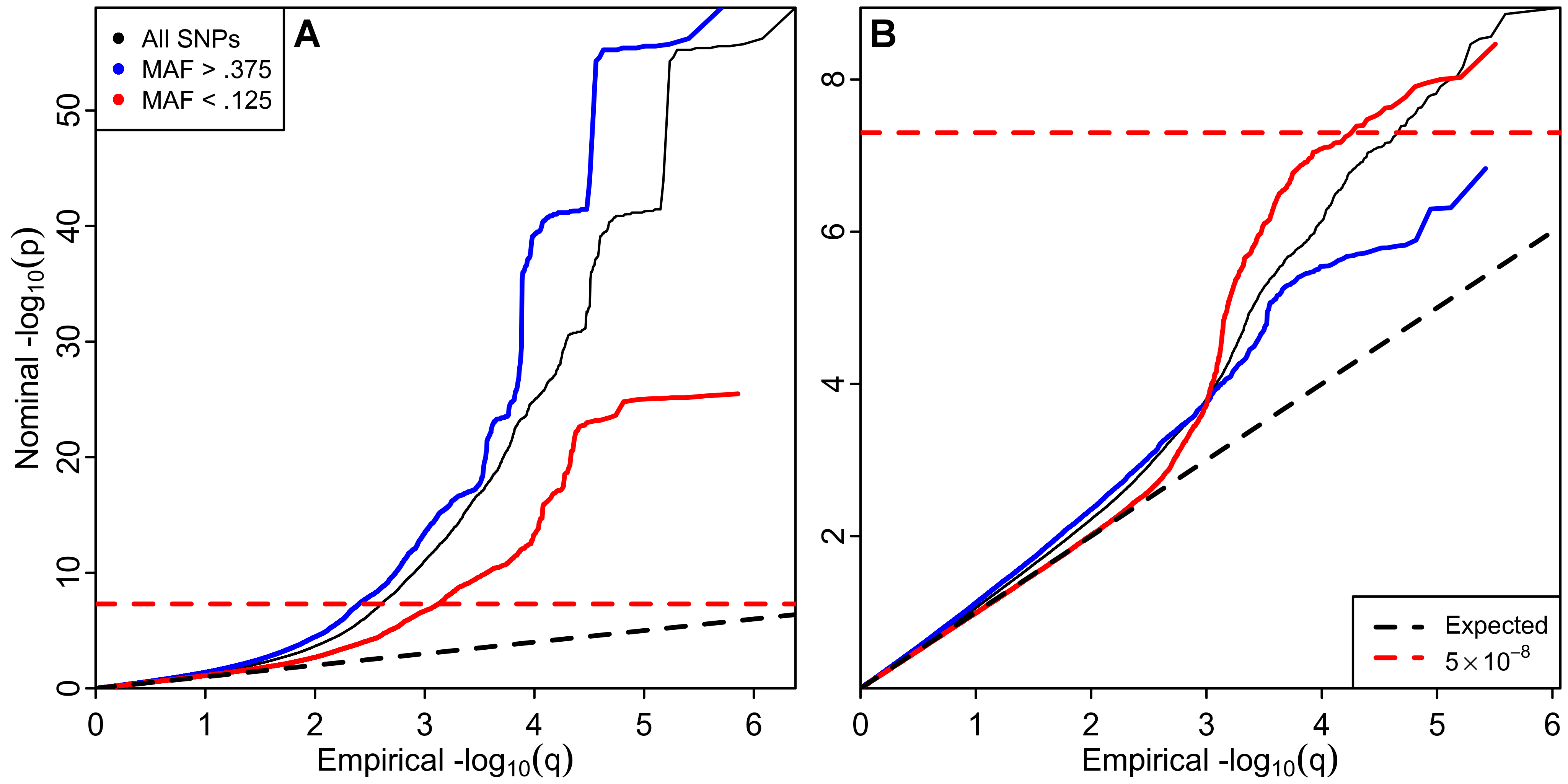

Supplement: Figure S7 — Enrichment by MAF category for height and schizophrenia. The effect of minor allele frequency (MAF) is not consistent across phenotypes. (A) For height more common SNPs show a continually larger enrichment than less common SNPs. (B) For Schizophrenia common SNPs show more enrichment at moderate to small z-scores, but for larger z-scores less common SNPs are more enriched. (TIF) [file pgen.1003449.s007.tif]

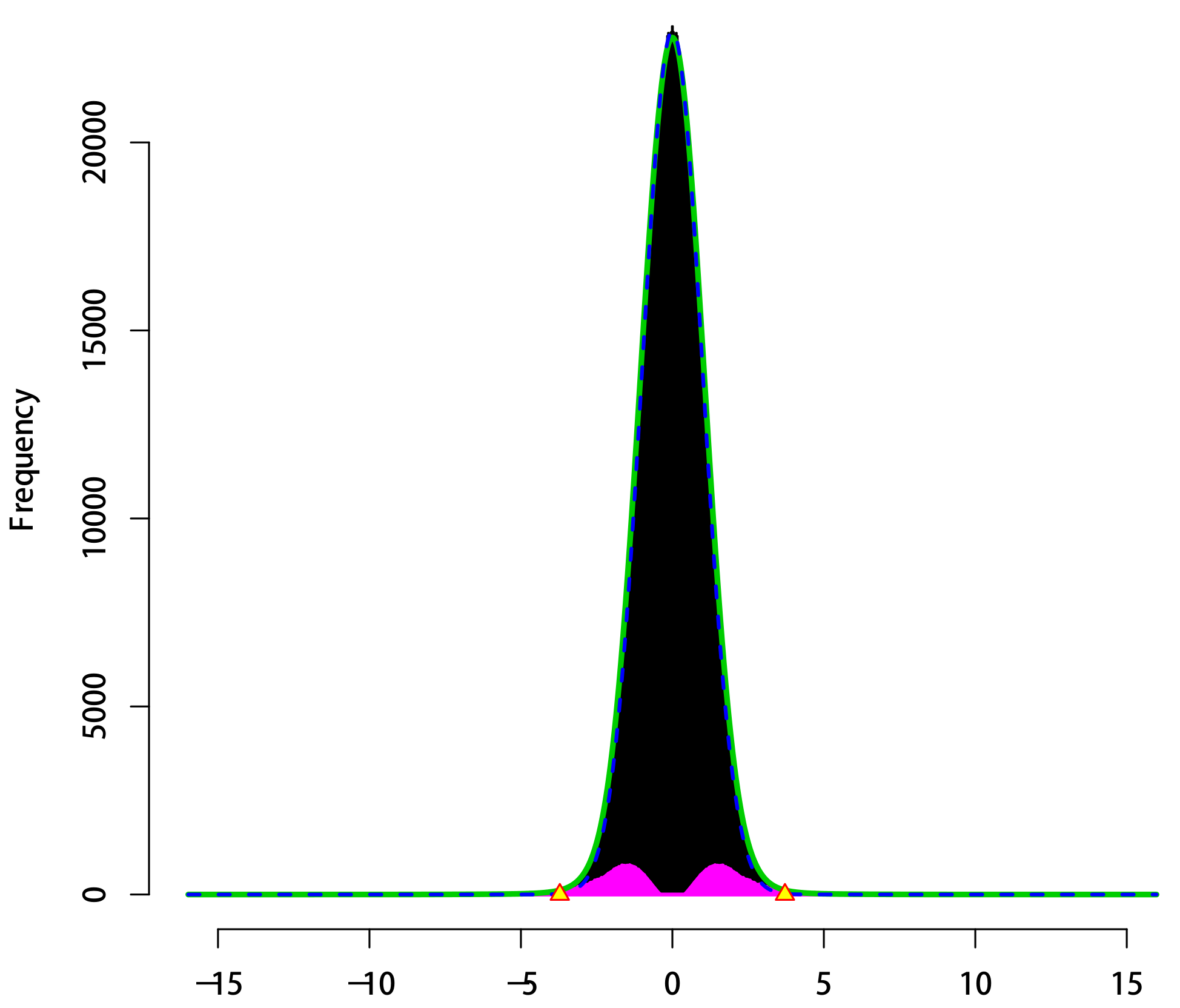

Supplement: Figure S8 — locfdr plot for all SNPs in Crohn's disease. Mixture model fits for all SNPs for Crohn's disease. Black: empirical z-score distribution, Purple: estimated non-null distribution, Green line: smooth estimate of mixture distribution (full distribution), Blue line: smooth estimate of the null distribution. Diamonds: local false discovery rate (LFDR) of 0.2. The estimated proportion of non-null SNPs varies by category, as does the variance in the estimated non-null effect size. (TIF) [file pgen.1003449.s008.tif]

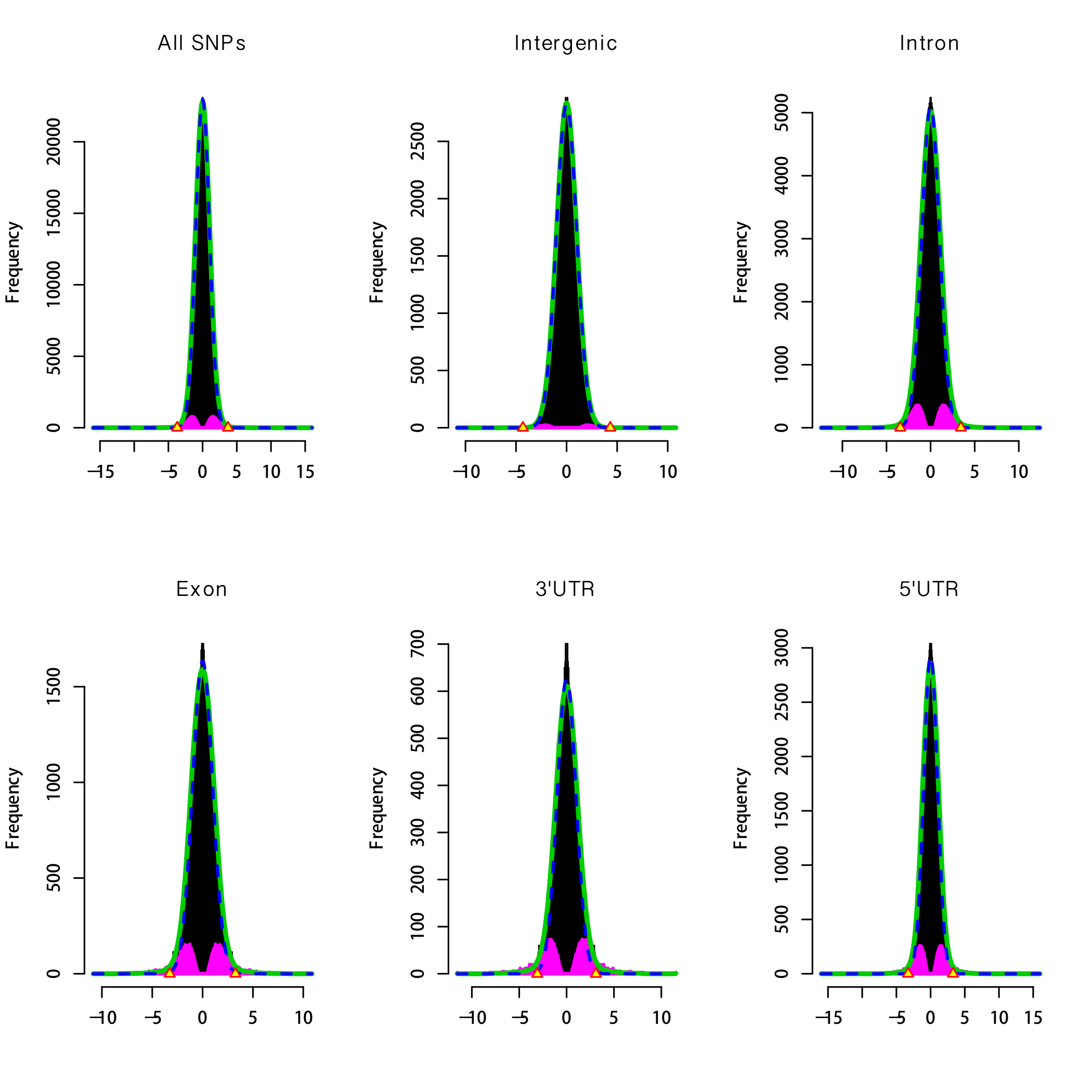

Supplement: Figure S9 — locfdr plots for genic annotation categories in Crohn's disease. Mixture model fits for each annotation category for Crohn's disease. Black: empirical z-score distribution, Purple: estimated non-null distribution, Green line: smooth estimate of mixture distribution (full distribution), Blue line: smooth estimate of the null distribution. Diamonds: local false discovery rate (LFDR) of 0.2. The estimated proportion of non-null SNPs varies by category, as does the variance in the estimated non-null effect size. (TIF) [file pgen.1003449.s009.tif]

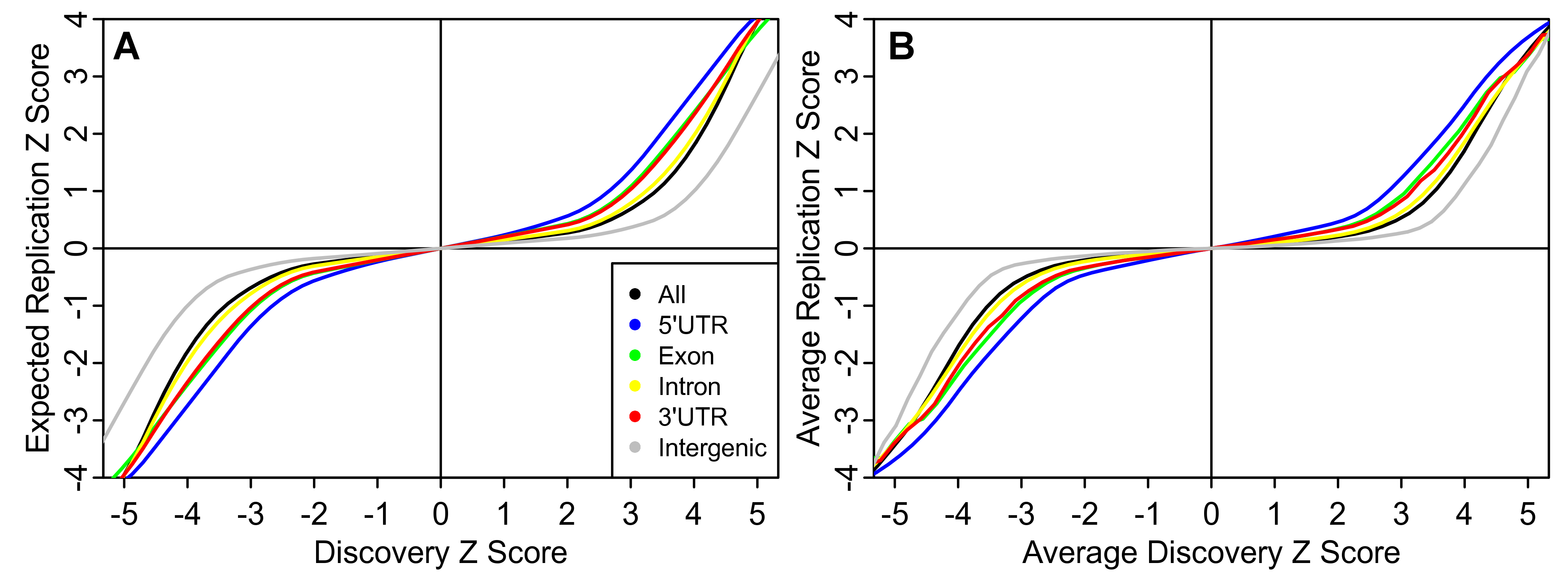

Supplement: Figure S10 — Z-score–z-score plots confirm mixture model predictions. (A) Expected a posteriori estimates of effect size for a given observed z-score. (B) Z-score-z-score plot demonstrates the empirical replication z-scores closely match the expected a posteriori effect sizes and are strongly dependent upon genic annotation category. (TIF) [file pgen.1003449.s010.tif]

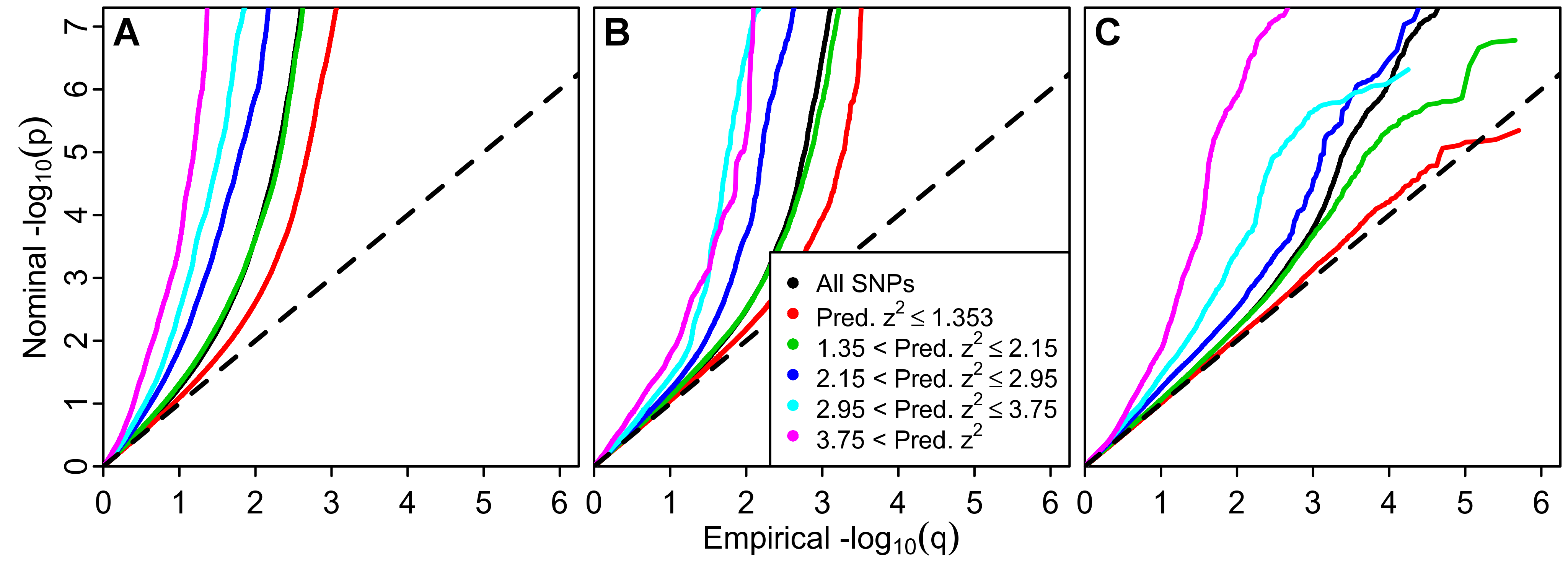

Supplement: Figure S11 — Regression based strata enrichment. Q-Q plot enrichment for the regression based strata for (A) Height, (B) Crohn's Disease (CD), and (C) Schizophrenia (SCZ). SNPs predicted to have a higher tagged variance (z2) show greater levels of enrichment. Enrichment is consistent across these three phenotypes, despite being determined from a regression model only using the summary statistics from the height GWAS. (TIF) [file pgen.1003449.s011.tif]

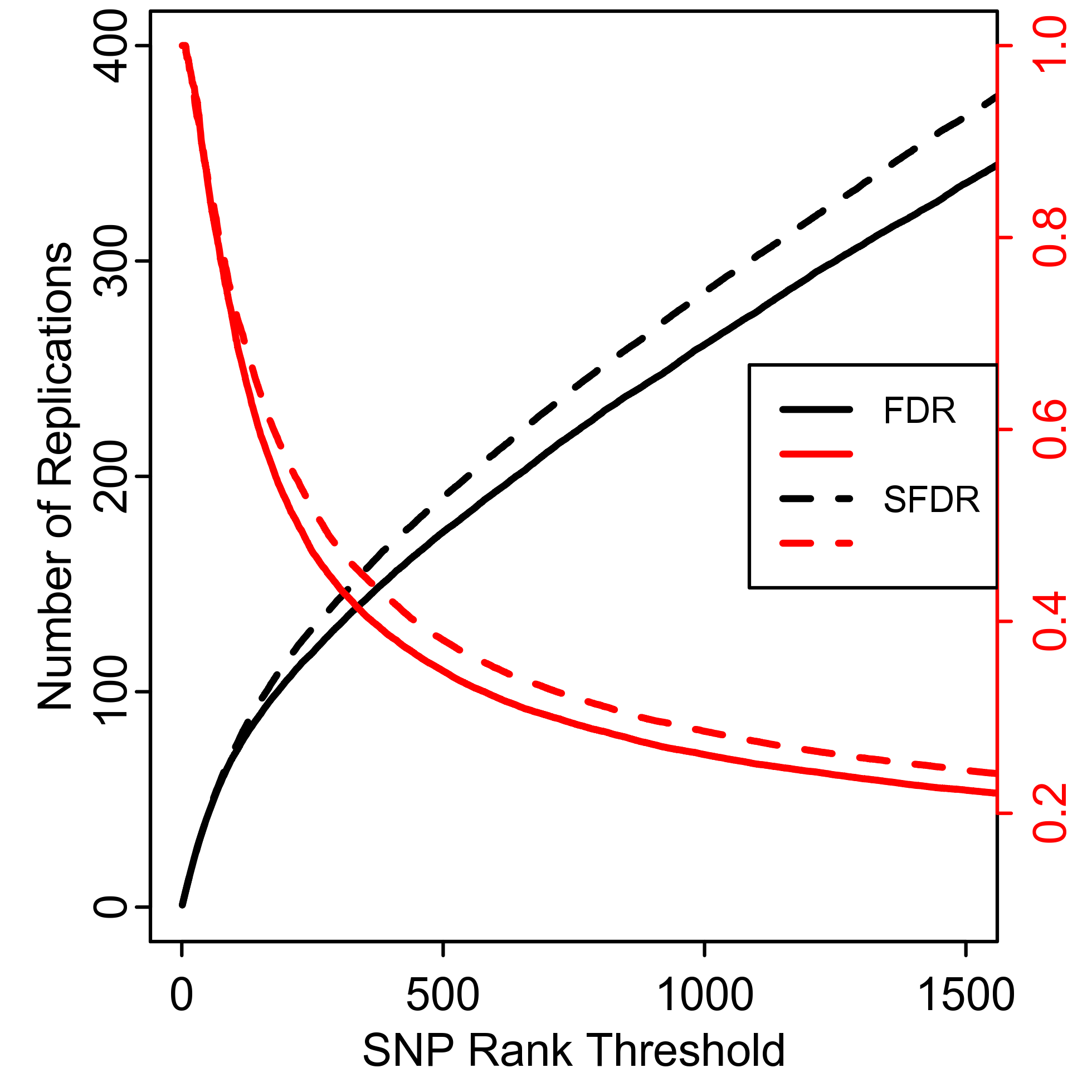

Supplement: Figure S12 — Replication Rates of sFDR versus FDR. For a given SNP rank threshold (i.e., top 500 SNPs), those ranked by the genic annotation category-informed stratified FDR show a greater absolute number of replications, and thus a greater rate of replication, when compared to the annotation un-informed standard FDR. When incorporated into an FDR framework, the enriched genic annotation categories lead to an increased rate of true discovery among SNPs at a given cut off. (TIF) [file pgen.1003449.s012.tif]

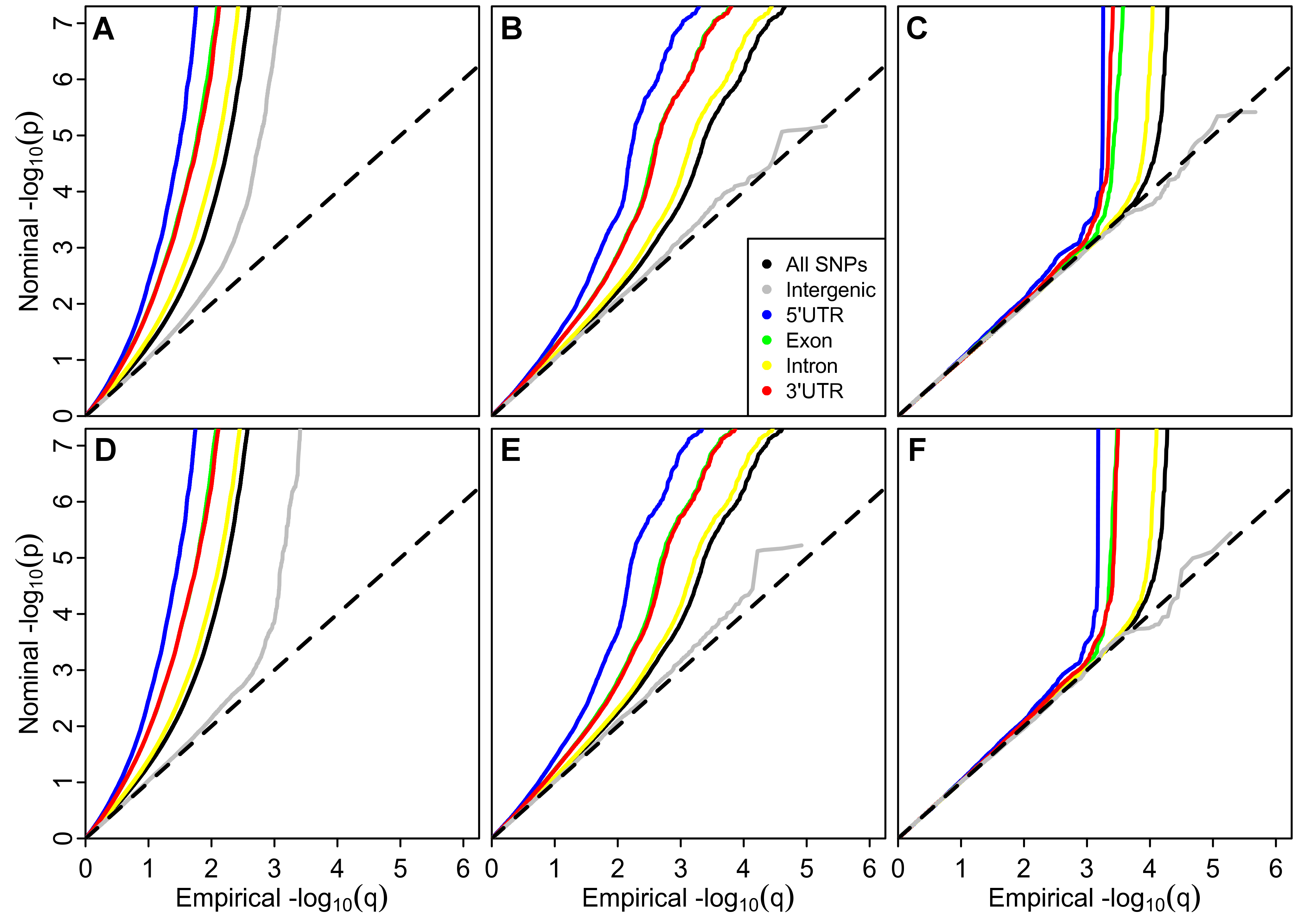

Supplement: Figure S13 — Stratified QQ-plots with different scoring parameters. The original stratified QQ-plots for height (A), Schizophrenia (B), and Cigarettes per day (C) using LD-weighted annotation categories created from an LD matrix describing the pairwise correlation between each GWAS SNP and all 1000 genomes SNPs (described above) including r2 values greater than 0.2 and within 1 megabase of the target GWAS SNP show a qualitatively similar pattern of enrichment when the scoring parameters are changed to include all pairwise r2 values greater than 0.05 and within 2 megabases (Height, D; Schizophrenia, E; Cigarettes per day, F). (TIF) [file pgen.1003449.s013.tif]

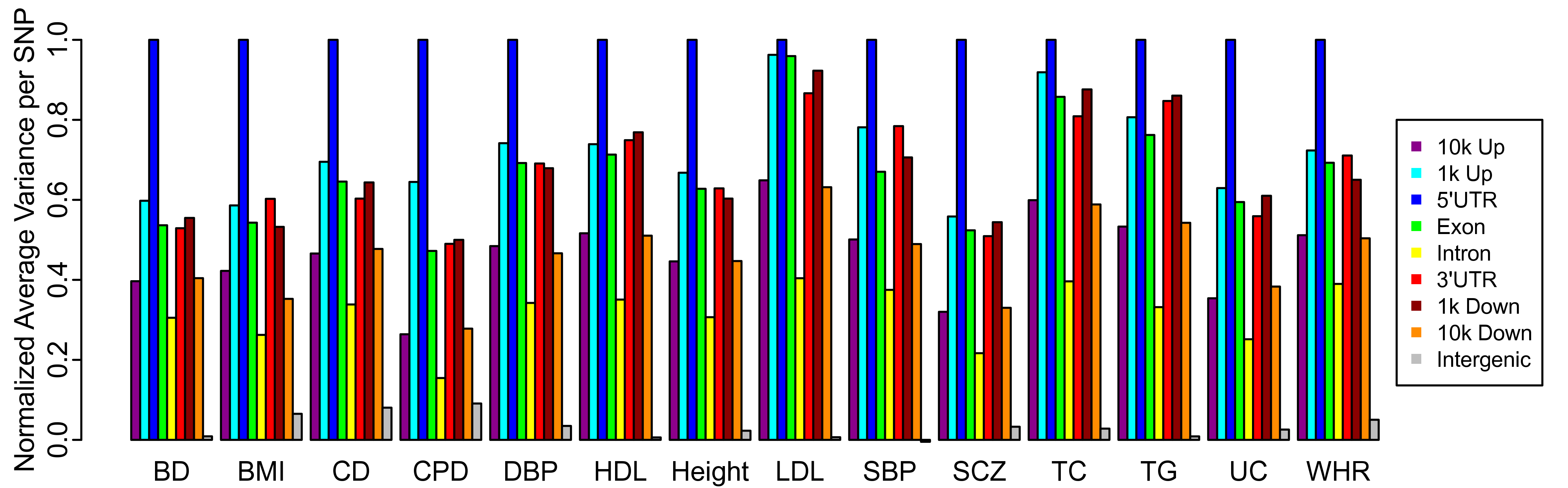

Supplement: Figure S14 — Mean(z-score2−1) plot with alternate scoring parameters. The patterns among the mean(z-score2−1) for each category of SNPs per phenotype is robust to LD-weighted annotation scoring parameters as the patterns match those shown in Figure S6. Here we show results when pairwise LD is thresholded at r2>0.05 and within 2 megabases (original scoring: r2>0.2 and within 1 megabase). BD, Bipolar Disorder; BMI, Body Mass Index; CD, Crohn's disease; CPD, Cigarettes per Day; DBP, Diastolic blood pressure; HDL, High density lipoprotein; LDL, Low density lipoprotein; SBP, systolic blood pressure; SCZ, Schizophrenia; TC, total Cholesterol; TG, triglycerides; UC, Ulcerative Colitis; WHR, Waist-hip-ratio. (TIF) [file pgen.1003449.s014.tif]

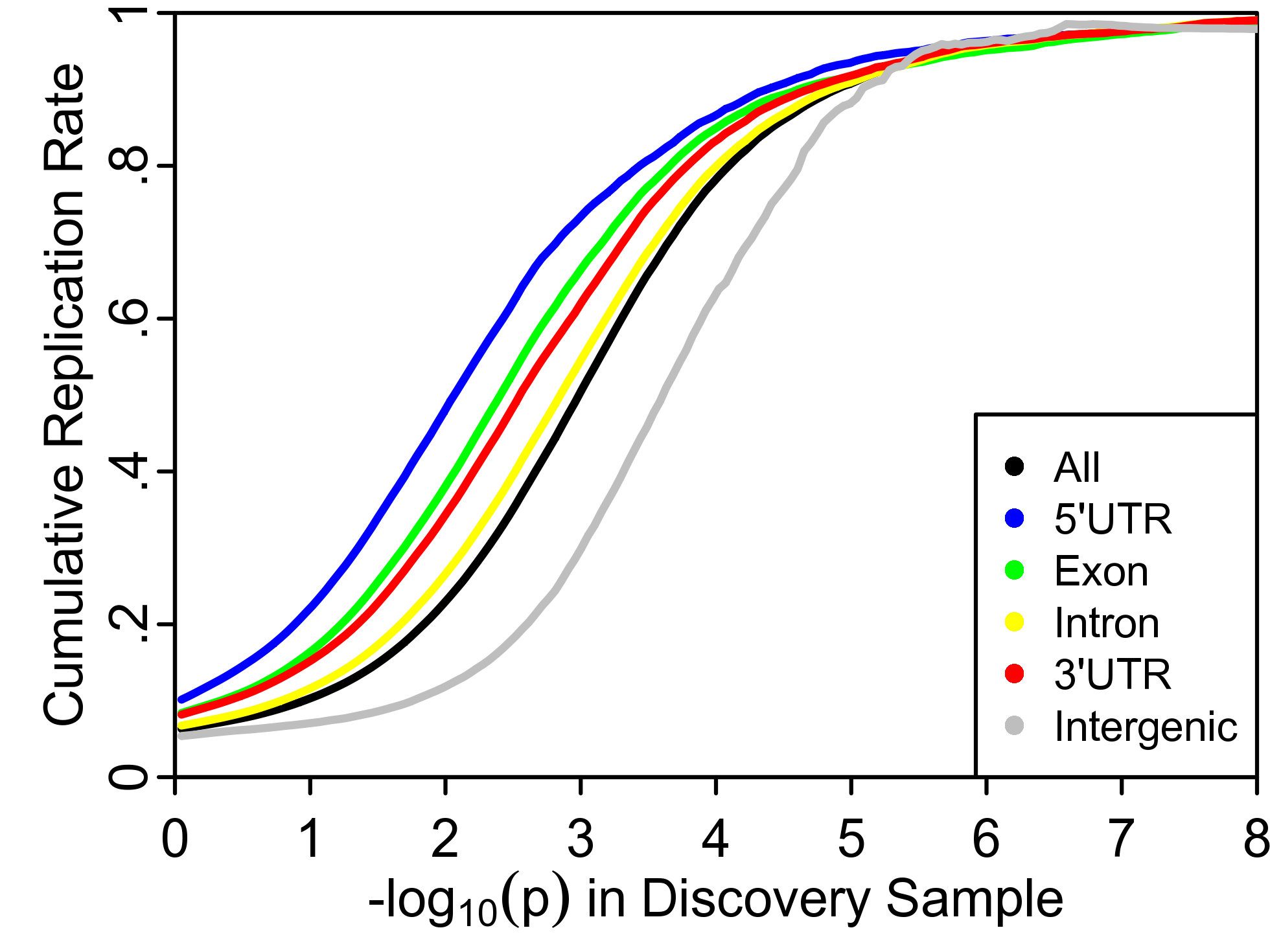

Supplement: Figure S15 — Replication rate among categories with alternate scoring parameters. A regenerated cumulative replication plot (Figure 4B) showing the average rate of replication (p<.05) within independent sub-studies for a given p-value. The enrichment produced by the alternate LD weighted annotation scoring parameters (including r2>0.05 and all SNPs within 2 megabases) results in a similar pattern of increased replication as with the original parameters (including r2>0.2 and all SNPs within 1 megabases), with the exception of the intergenic category, which shows a noticeable decrease in the replication rate. (TIF) [file pgen.1003449.s015.tif]

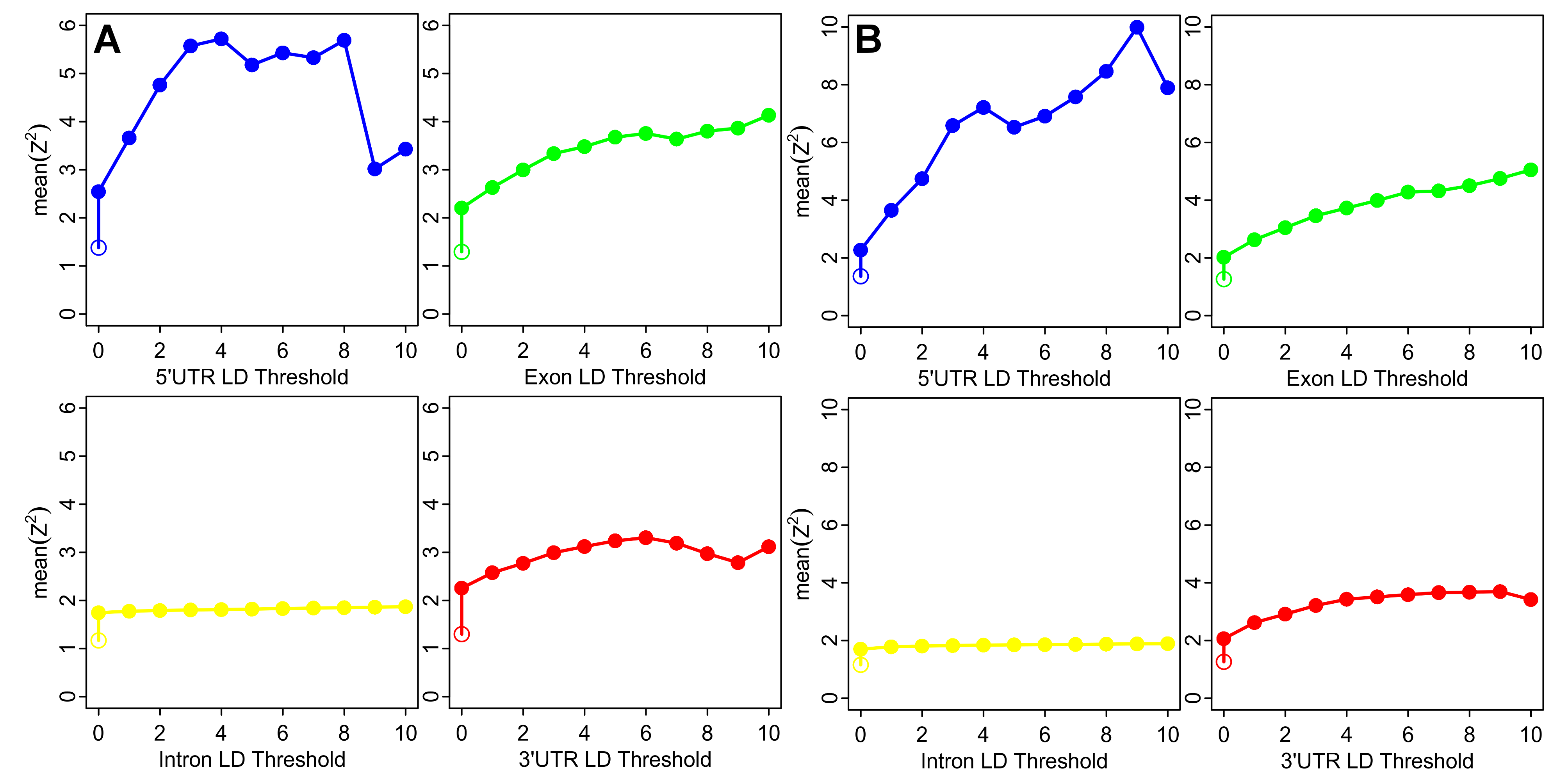

Supplement: Figure S16 — Relationship between total categorical total LD and z-score2. The mean (z2) of each category, using the height GWAS, as we change the threshold for inclusion for both the original (A; including r2>0.2 and within 1 megabases), and alternate (B; r2>0.05 and within 2 megabases) parameters for LD weighted scoring. The mean(z2) increases approximately monotonically each category, but with noticeably different slopes. The 5′UTR category in figure A becomes unstable at high thresholds because there are very few SNPs remaining. Changing to a more inclusive LD weighted scoring increases the number of SNPs with high scores and improves the relationship. This suggests that even greater enrichment could be achieved by tuning the categorical inclusion threshold upwards. (TIF) [file pgen.1003449.s016.tif]

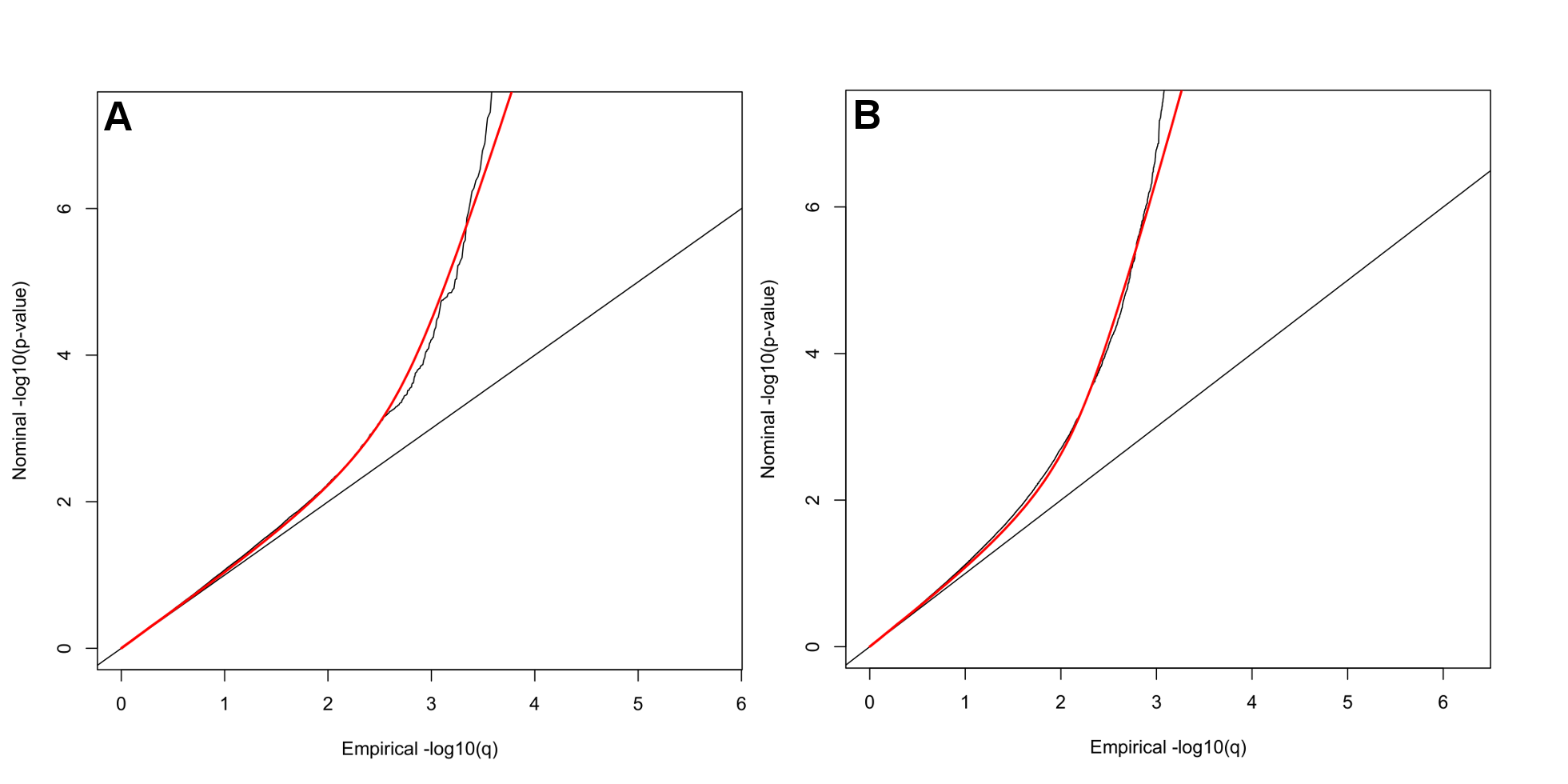

Supplement: Figure S17 — Parametric mixture model fits to Q-Q plots. Q-Q Plot for Height (A) and Crohn's Disease (B). Solid black lines are actual data. Dotted black lines are Q-Q curves under the global null hypothesis. Solid red lines are fitted Q-Q curves from Weibull mixture model for transformed p-values. Note, upper limit in Q-Q plot y-axes is 7.3, corresponding to GWAS-significance threshold of p = 5×10^−8^. (TIF) [file pgen.1003449.s017.tif]

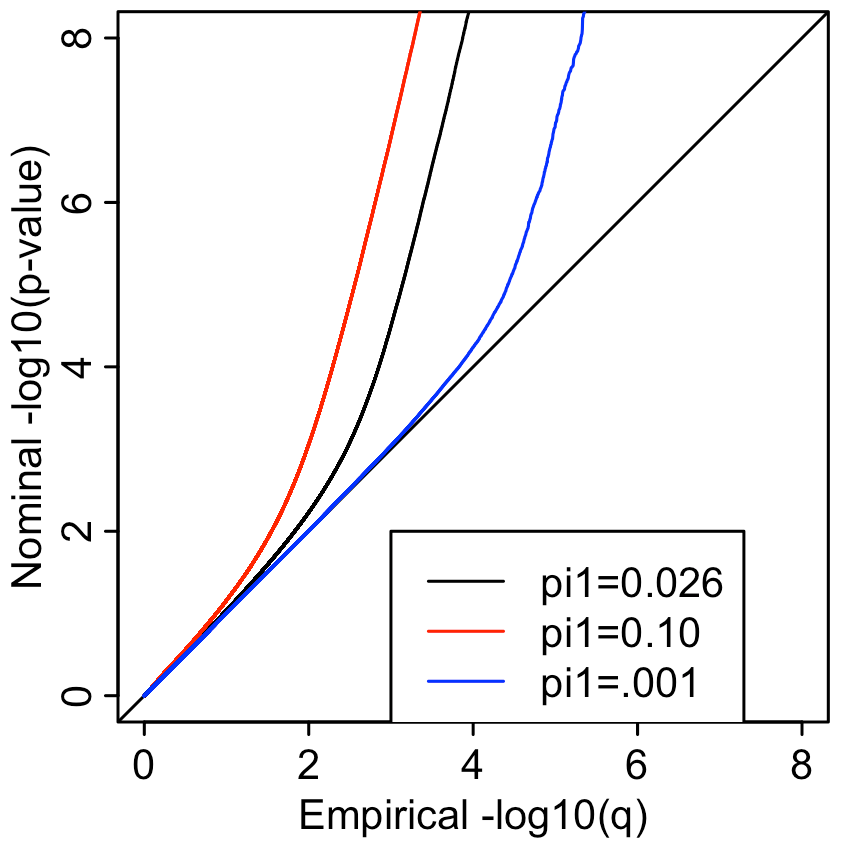

Supplement: Figure S18 — Effect of non-null proportion on Q-Q plots. Predicted Q-Q Plot for Crohn's Disease (CD; solid black line) from parametric Weibull mixture model fit (model given by Equation [S9]). The blue line is the predicted Q-Q curve of the CD data if the non-null proportion π1 were 0.001 instead of the value 0.026 estimated from the CD data. The red line is the predicted Q-Q curve if the non-null proportion π1 were 0.10. (TIF) [file pgen.1003449.s018.tif]

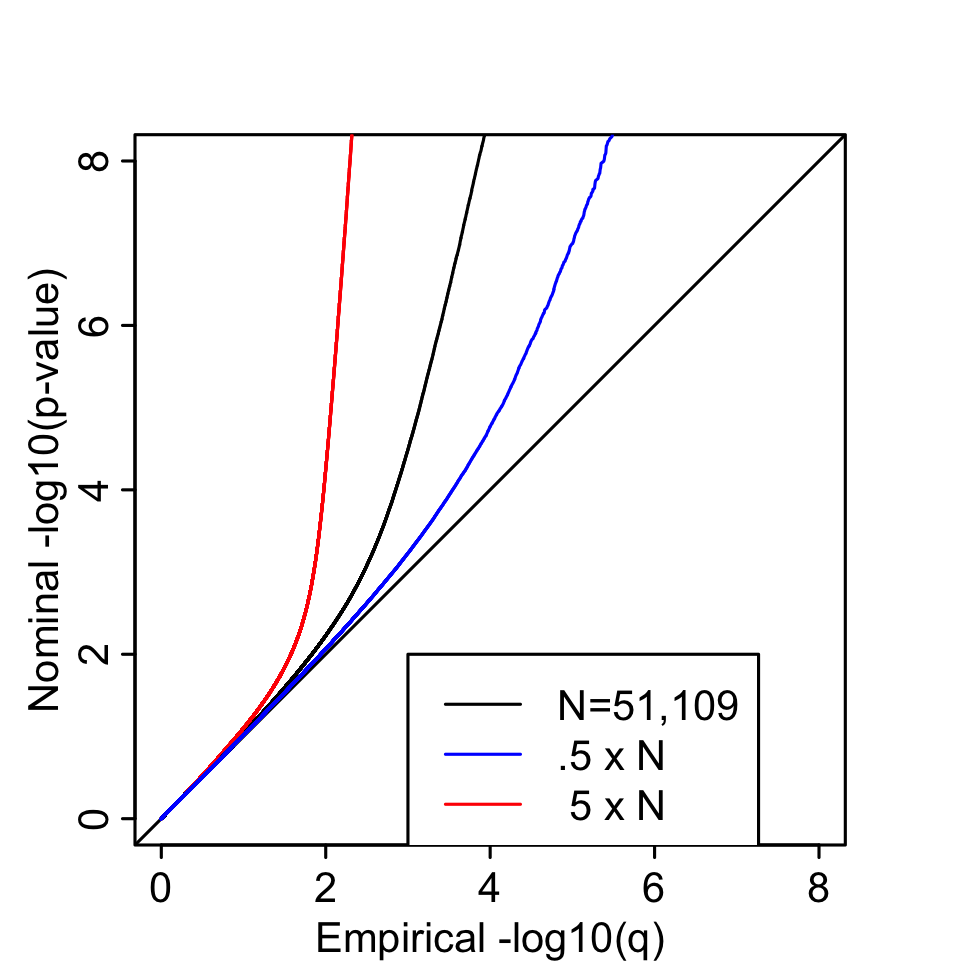

Supplement: Figure S19 — Effect of sample size on Q-Q plots. Predicted Q-Q Plot for Crohn's Disease (CD; solid black line) from parametric Weibull mixture model fit (model given by Equation [S9]). The blue line is the predicted Q-Q curve of the CD data if the sample size were half as large as the true sample size (n = 51,109). The red line is the predicted Q-Q curve of the CD data if the sample size were five times as large as the true sample size. (TIF) [file pgen.1003449.s019.tif]
