# Supplementary material for: All SNPs Are Not Created Equal: Genome-Wide Association Studies Reveal a Consistent Pattern of Enrichment among Functionally Annotated SNPs
Source: PLoS Genet. 2013 Apr 25;9(4):e1003449. doi: 10.1371/journal.pgen.1003449 (PMC3636284; doi:10.1371/journal.pgen.1003449)
Supplement: Table S10 — Multiple regression analysis predicting log(Z2) in height. A multiple regression analysis reveals a minimal, but significant, effect of total LD on the log z2 for height. This represents a minimal, but significant, effect of overall LD block size on enrichment. Categorical effects remain independently strong in this analysis with an effect size order that mirrors enrichment. (DOCX) [file pgen.1003449.s029.docx]

| **Variables** | **Coeff.** | **Adjusted SE*** | **Adjusted 95% CI*** |
| --- | --- | --- | --- |
| Intercept | -1.2027 | 0.00108 | (-1.2048, -1.2006) |
| Total LD | 0.0019 | 0.00008 | (0.0018, 0.0021) |
| Intron | 0.0025 | 0.00013 | (0.0022, 0.0028) |
| Exon | 0.1686 | 0.00543 | (0.0062, 0.0275) |
| 3’UTR | 0.1182 | 0.0044 | (0.1182, 0.1269) |
| 1K Upstream | 0.0905 | 0.00668 | (0.0774, 0.1035) |
| 5’UTR | 0.3467 | 0.01303 | (0.3212, 0.3723) |

*Standard errors of regression coefficients adjusted to reflect effective independent sample size degrees of freedom of 10^5.
