# Supplementary material for: All SNPs Are Not Created Equal: Genome-Wide Association Studies Reveal a Consistent Pattern of Enrichment among Functionally Annotated SNPs
Source: PLoS Genet. 2013 Apr 25;9(4):e1003449. doi: 10.1371/journal.pgen.1003449 (PMC3636284; doi:10.1371/journal.pgen.1003449)
Supplement: Table S11 — Null GWAS Simulations. We present simulations of categorical enrichment based on multiple independent null GWAS simulations using subjects with European ancestry from the 1000 Genomes Project. Random phenotypes were generated unrelated to genotypes for each subject, association z-scores were computed for each tag SNP, and mean(z2) was computed for each annotation category, using the same procedure as applied to the actual GWAS data. The means and standard deviations were computed from 20 independent simulation runs. The results demonstrate that the observed differential enrichment of annotation categories cannot be explained by category-specific spurious sources of genomic inflation due to differential LD or MAF. (DOCX) [file pgen.1003449.s030.docx]

| **Annotation category** | **mean z^2^** | **stdev** |
| --- | --- | --- |
| 10kUp | 0.997 | -0.014 |
| 1kUp | 0.996 | -0.018 |
| 5'UTR | 1.003 | -0.033 |
| Exon | 1 | -0.021 |
| Intron | 0.998 | -0.013 |
| 3'UTR | 1.001 | -0.016 |
| 1kdown | 0.994 | -0.015 |
| 10kDown | 1 | -0.013 |
| Intergenic | 0.999 | -0.018 |
