# Supplementary material for: All SNPs Are Not Created Equal: Genome-Wide Association Studies Reveal a Consistent Pattern of Enrichment among Functionally Annotated SNPs
Source: PLoS Genet. 2013 Apr 25;9(4):e1003449. doi: 10.1371/journal.pgen.1003449 (PMC3636284; doi:10.1371/journal.pgen.1003449)
Supplement: Text S1 — Supplementary text extending Materials and Methods and presenting supporting analyses. More details are provided with respect to the acquisition, processing and annotating of the GWAS data used for the main results. The relationship between QQ-plots and False Discovery Rate is extended and related to our measures of enrichment. Also, the main results are described within the context of a mixture-modeling framework. Finally, a series of control experiments are described and supplementary references are enumerated. (DOCX) [file pgen.1003449.s032.docx]

**Text S1**

| **Contents** | **Page** |
| --- | --- |
| Data Acquisition and Processing | 2 |
| Genic Annotation Categories | 3 |
| Linkage Disequilibrium (LD) Weighted Annotation Score | 5 |
| Intergenic Inflation Control | 7 |
| Q-Q Plots and False Discovery Rate (FDR) | 8 |
| Quantification of Enrichment | 10 |
| Consistency with Local False Discovery Rate Estimation | 11 |
| A Parametric Mixture Model | 12 |
| Control for Potential Confounds: LD and MAF | 14 |
| Replication Estimates | 15 |
| Robustness of LD Weighted Scoring Procedure | 16 |
| Supplementary References | 19 |

**Data Acquisition and Processing**

For all studies, Genome-wide association study (GWAS) results in the form of summary statistic p-values were obtained from public access websites[1-4], published online supplementary material[5] or through collaboration with investigators[6-9]. For Crohn’s disease (CD)[6] pre-meta-analysis, sub-study specific p-values and effect sizes (z-scores) were obtained from the study principal investigators. See Table S1.

In total we consider over 1.3 million phenotypic observations; however, due to considerable overlap in samples, the number of unique individuals surveyed is significantly less. Blood pressure phenotypes (systolic blood pressure; SBP, diastolic blood pressure; DBP) were a part of one study sample[5] as were lipid traits (triglycerides; TG, total Cholesterol; TC, High density lipoprotein; HDL, Low density lipoprotein; LDL)[4]. In addition, Body Mass Index (BMI)[1], Height[2] and Waist-hip-ratio (WHR)[3] *all* arose from the GIANT consortium and there is thus much sample redundancy.

The samples used in the lipids GWAS[4] overlap considerably with the GIANT consortium samples, as do the samples used in the smoking GWAS[10]. The Schizophrenia[8] and Bipolar Disorder GWAS[9] share some controls. Without genotype data it is impossible to fully characterize the nature of this overlap, but we note that it is considerable and likely extends beyond what is listed here. The phenotypes, however, are diverse. Also, if shared controls among case-control studies were a confound in our analyses it would imply that the original study results were driven by this confound as well, and not by true trait associations. Overlap is unlikely to drive our results across phenotypes. The stringently defined independent sample replications (Figure 3) for CD confirm that shared controls are not explaining the observed results.

**Genic Annotation Categories**

Bi-allelic SNP genotypes from the European reference sample provided by the November 2010 release of Phase 1 of the 1000 Genomes Project (1KGP) were obtained in pre-processed form from <http://www.sph.umich.edu/csg/abecasis/MACH/download/>. We performed additional quality control on the 1KGP data using Plink version 1.07[11,12]. 1KGP genotypes were pruned according to standard GWAS procedures, removing all SNPs with a minor allele frequency less than 1%, missing in more than 5% of individuals or violating Hardy-Weinberg equilibrium (p < 1x10^-6^). Individuals missing more than 10% of genotypes were excluded. Plink implementations of identity by state (IBS) and identity by descent (IBD) analysis were used to remove one individual from each related pair present and implementations of multidimensional scaling were used to ensure population homogeneity within our reference sample.

Each SNP in the 1KGP based reference sample was assigned a mutually exclusive category based on its position within the genome. We leveraged a computational annotation pipeline[13] which calls upon a variety of publicly available tools and databases to aggregate comprehensive functional and positional information for any one variant. For variants in genes with multiple transcripts or at positions that correspond to multiple genes categories were assigned based only on the position within the first gene listed in the UCSC known genes database[14]. In total 9,078,405 1KGP SNPs were annotated with positional categories. All positional categories were scored 0 or 1.

The following genic annotation categories were used:

*10k Up.* This category consisted of all 1KGP SNPs that were between 10,000 and 1,001 base pairs upstream of the transcription start site for the primary listing of protein coding genes in the UCSC known genes database[14]. For SNPs gene dense areas, priority was given to upstream category over downstream. Thus SNPs both 10,000 base pairs upstream and downstream from a protein coding gene were only annotated with the upstream category.

*1k Up.* This category consisted of all 1KGP SNPs that were between 1,000 and 1 base pair(s) upstream of the transcription start site for the primary listing of protein coding genes in the UCSC known genes database[14]. For SNPs gene dense areas, priority was given to upstream category over downstream. Thus SNPs both 1,000 base pairs upstream and downstream from a protein coding gene were only annotated with the upstream category.

*5’UTR.* This category consisted of all 1KGP SNPs that were located within the five prime untranslated region (5’UTR) of the primary listing of protein coding genes in the UCSC known genes database[14]. All regions that are transcribed, but not translated, are assigned to UTR categories. If a polymorphism was within an exon or intron within a 5’UTR, it was annotated only as 5’UTR.

*Exon.* This category consisted of all 1KGP SNPs that were located within an exon of the primary listing of protein coding genes in the UCSC known genes database[14]. If a polymorphism was within an exon that fell within the 5’UTR or 3’UTR of a gene, it was annotated only as 5’UTR or 3’UTR.

*Intron.* This category consisted of all 1KGP SNPs that were located within an intron of the primary listing of protein coding genes in the UCSC known genes database[14]. If a polymorphism was within an intron that fell within the 5’UTR or 3’UTR of a gene, it was annotated only as 5’UTR or 3’UTR.

*3’UTR.* This category consisted of all 1KGP SNPs that were located within the three prime untranslated region (3’UTR) of the primary listing of protein coding genes in the UCSC known genes database[14]. All regions that are transcribed, but not translated, are assigned to UTR categories. If a polymorphism was within an exon or intron within a 3’UTR, it was annotated only as 5’UTR.

*1k Down.* This category consisted of all 1KGP SNPs that were between 1 and 1,000 base pair(s) downstream of the transcription start site for the primary listing of protein coding genes in the UCSC known genes database[14]. For SNPs gene dense areas, priority was given to upstream category over downstream. Thus SNPs both 1,000 base pairs upstream and downstream from a protein coding gene were only annotated with the upstream category.

*10k Down.* This category consisted of all 1KGP SNPs that were between 1,001 and 10,000 base pair(s) downstream of the transcription start site for the primary listing of protein coding genes in the UCSC known genes database[14]. For SNPs gene dense areas, priority was given to upstream category over downstream. Thus SNPs both 10,000 base pairs upstream and downstream from a protein coding gene were only annotated with the upstream category.

Additional categories were recorded, including 10,001 – 100,000 BP up and downstream of protein coding genes, presence within a non-coding RNA, presence within a transcription factor binding site, and presence within a microRNA binding site. These categories were used to help select intergenic SNPs but were not analyzed in terms of differential enrichment (see discussion below).

**Linkage Disequilibrium (LD) Weighted Annotation Score**

We leveraged the above positional annotations in the densely mapped 1KGP to characterize the types of variants that each GWAS studied SNP was a surrogate for, or tagged, as a result of Linkage Disequilibrium (LD). Each GWAS performed quality control according to best practices, as describes in detail in each of the original publications[1-10]. We did not perform additional quality control measures on GWAS summary statistics beyond intergenic inflation correction (described below). GWAS SNPs with reference SNP (rs) numbers that did not map to the 1KGP were excluded, as we could not determine the LD structure within the 1KGP reference panel.

In order to assign LD-weighted annotation scores, a correlation coefficient approximation to r^2^ pairwise linkage disequilibrium (LD) was calculated using Plink version 1.07[11,12]. For each GWAS tag SNP present in the 1KGP pairwise LD was calculated to all other 1KGP SNPs within 1,000,000 base pairs (1Mb) on either side of the SNP. This provided, for each SNP, a 2Mb window in which LD scores were considered. LD scores were thresholded at r^2^ ≥ 0.2. LD scores were continuous valued from 0.2 to 1. Each SNP was assigned an LD value of 1 with itself (The robustness of our results to these parameter settings is discussed below in the section entitled *Robustness of LD Weighted Scoring Procedure*).

For each GWAS tag SNP, continuous, non-exclusive LD-weighted category scores were assigned as the LD weighted sum of the positional category scores for variants tagged in each of the eight categories mentioned above as annotated in the 1KGP reference panel. Summary statistics describing the distribution of scores in each category for the 2,558,411 SNPs representing the union of all GWAS considered are provided in Table S2.

Intergenic SNPs were determined after LD-weighted scoring. They were defined by weighted LD scores for each of the eight categories equal to zero. In addition these SNPs did not tag any SNPs in the 1KGP reference panel located within 100,000 base pairs of a protein coding gene, within a noncoding RNA, within a transcription factor binding site nor within a microRNA binding site.

For comparison and to assess the effect of leveraging LD weighted scoring in this way comparisons were made between LD-weighted scores (Figure 1) and positional or non-LD-weighted scores (i.e., using the categories of the tag SNPs themselves, and ignoring the annotation categories of SNPs in LD with the tag SNP, Figure S3). Continuous valued scores were turned into binary categories by thresholding scores at a lower bound for inclusion of 1.0. SNPs with a score less than 1 were not counted as a category member. A schematic of our scoring method is presented in Figure S1. Counts of SNPs in each category based on LD-weighted and non-LD-weighted (1KGP position only) are tabulated in Table S3.

**Intergenic Inflation Control**

The empirical null distribution in GWAS is affected by global variance inflation due to population stratification and cryptic relatedness[15] and deflation due to over-correction of test statistics for polygenic traits[16] by standard genomic control methods. We applied a control method leveraging only intergenic SNPs that are likely depleted for true associations. All p-values were converted into z-scores, and, for each phenotype, the genomic inflation factor[15], λ_GC_, was estimated for intergenic SNPs. All test statistics were divided by λ_GC_. The inflation factor, λ_GC_ was computed as the median z-score squared divided by the expected median of a chi-square distribution with one degree of freedom for all phenotypes except CPD, where the .95 quantile was used in place of the median. For correction statistics see Table S4.

We leveraged the intergenic SNPs to estimate inflation because their relative depletion of associations suggests they provide a robust estimate of true null SNPs that is uncontaminated by polygenic effects. Using annotation categories in this fashion is important given concerns posed by recent GWAS[8] about the over-correction of test statistics using standard genomic control. We present statistics from this procedure in Table S4. We report the traditional GC value for the summary statistics from each GWAS in their received state. Original values less than 1.0 suggest an over correction by traditional GC metrics, while values greater than 1.0 suggest an under correction or no correction at all. The values that remain after intergenic inflation correction are likely to represent variance inflation due to true polygenic effects.

**Q-Q Plots and False Discovery Rate (FDR)**

Q-Q plots are standard tools for assessing similarity or differences between two cumulative distribution functions (cdfs)[17]. When the probability distribution of GWAS summary statistic two-tailed p-values is of interest, under the global null hypothesis the theoretical distribution is uniform on the interval [0,1]. If nominal p-values are ordered from smallest to largest, so that p_(1)_ < p_(2)_ < … < p_(N)_, the corresponding empirical cdf, denoted by “q”, is simply q_(i)_ = i/N (in practice adjusted slightly to account for the discreteness of the empirical cdf), where N is the number of SNPs in the GWAS (or genic category). Thus, for a given index i, the x-coordinate of the Q-Q curve is simply q_(i)_, since the theoretical inverse cdf is the identity function, and the y-coordinate is simply the nominal p-value p_(i)_. As is common practice in GWAS, we plot instead -log_10_ p against the -log_10_ q to emphasize tail probabilities of the theoretical and empirical distributions; these coordinates are labeled “nominal –log_10_ (p)” and “empirical –log_10_ (q)” in the Q-Q plots. For a given threshold of GC-controlled p-values, category ‘enrichment’ is seen as a horizontal (not vertical) deflection of the Q-Q curves from the identity line (or from one genic category to another) as described in detail next.

The ‘enrichment’ seen in the Q-Q plots can be directly interpreted in terms of False Discovery Rate (FDR)[18]. For a given p-value cutoff, the Bayes FDR **[19]** is defined as

FDR(p) = π_0_F_0_(p) / F(p), [S1]

where π_0_ is the proportion of null SNPs, F_0_ is the null cumulative distribution function (cdf), and F is the cdf of all SNPs, both null and non-null; see below for details on this simple mixture model formulation[20]. Under the null hypothesis, F_0_ is the cdf of the uniform distribution on the unit interval [0,1], so that Eq. [S1] reduces to

FDR(p) = π_0_ p / F(p), [S2]

The cdf F can be estimated by the empirical cdf q = N_p_ / N, where N_p_ is the number of SNPs with p-values less than or equal to p, and N is the total number of SNPs. Replacing F by q in Eq. [S2], we get

FDR(p) ≈ π_0_ p / q, [S3]

which is biased upwards as an estimate of the FDR[20]. Replacing π_0_ in Equation [S3] with unity gives an estimated FDR that is further biased upward;

FDR(p) ≈ p / q [S4]

If π_0_ is close to one, as is likely true for most GWAS, the increase in bias from Eq. [S3] is minimal. The quantity 1 – p/q, is therefore biased downward, and hence a conservative estimate of the True Discovery Rate (TDR, equal to 1-FDR). Given the -log_10_ of the Q-Q plots we can easily read off

-log_10_(FDR(p) ) ≈ log_10_(q) – log_10_(p) [S5]

demonstrating that the (conservatively) estimated FDR is directly related to the horizontal shift of the curves in the Q-Q plots from the expected line x = y, with a larger shift corresponding to a smaller FDR. As before, the estimated true discovery rate can be obtained as one minus the estimated FDR. For each TDR plot in Figure 2 we calculated the TDR, using each observed p-value as a threshold, according to Eq. [S5].

**Quantification of Enrichment**

After appropriate genomic control enrichment can be assessed by its genic category-specific TDR for a given z-score (equivalently, nominal p-value). Categories of SNPs that have a higher TDR for a given nominal p-value are more “enriched” than categories of SNPs with a lower TDR for the same nominal p-value. This measure of enrichment depends on choice of p-value threshold.

An overall single number summary of category-specific enrichment is the sample mean of z^2^ minus one, where the mean is taken over all SNP z-scores in the given category. Both the TDR and the mean (z^2^)-1 are justified as measures of enrichment based on a simple Bayesian mixture model framework[20] . Specifically, let f(z) be the probability density for the SNP summary statistic z-scores. This is modeled as the mixture of a null probability density f_0_ and a non-null density f_1_

f(z) = π_0_f_0_(z) + π_1_f_1_(z)*,* [S6]

where, as above, π_0_ is the proportion of SNPs with no association with the trait and π_1_=1-π_0_ the proportion of SNPs with a non-zero association with the trait. Assuming that the z-scores are symmetric about zero, the variance of this distribution is

∫ z^2^ f(z) dz = ∫ z^2^ π_0_f_0_(z) dz + ∫ z^2^ π_1_f_1_(z) dz = π_0_ + π_1_∫ z^2^ f_1_(z) dz, [S7]

since the variance of the null distribution is one after appropriate genomic control. Under the assumption that the proportion of null SNPs (π_0_) is close to one, a mildly conservative estimate of the excess in variance attributable to non-null SNPs is given by ∫ z^2^ f(z) dz -1. An unbiased estimate of this expression is the sample mean of z^2^ minus 1. Note, non-null z-scores are scaled by the square root of the sample size, and hence mean(z^2^)-1 is proportional to, not identical with, π_1_ times the tagged phenotypic variance of the non-null SNPs.

**Consistency with Local False Discovery Rate Estimates**

Under scenarios of multiple testing, such as GWAS, quantitative estimates of likely true associations can be estimated from the distributions of summary statistics. Efron[19] has developed a flexible framework for quantitatively estimating the null, non-null and mixture distributions from the resulting test statistics. Similar approaches have been applied in other fields, most relevantly to gene expression array data[21] and linkage analysis[22]. As a demonstration we have fit the CD statistics using this model (Figures S8 and S9).

The empirical Bayesian modeling approach described by Efron[19] is implemented in the freely available R package *locfdr*[23]. The approach is to model the mixture density of effects in terms of z-scores as in Eq. [S6] above, or as a mixture density consisting of a weighted linear combination of a null density *f_0_(z)* for the z-scores of SNPs with no association, and a non-null density *f_1_(z)* for z-scores from trait-associated SNPs. The local false discovery rate (locfdr) is then given by

locfdr(z) = π_0_f_0_(z) / f(z)*,* [S8]

where f(z) is given by Eq. [S6]. Using this model, we estimated the empirical null density (assumed to be normal, with mean 0 and data determined standard deviation). We estimated the null for intergenic SNPs and adjusted all statistics accordingly such that the intergenic test statistics conformed to the theoretical distribution (normal with mean 0 and standard deviation 1). This approach mirrors our intergenic inflation control described previously. We then used the *locfdr* library to estimate the mixture density [1], fixing the null distribution to the theoretical standard normal and estimating the mixture density non-parametrically as a smoothed histogram. This model was fit to the overall data and per category (Figures S8 and S9).

This framework also allows us to estimate the *a posteriori* expected z-scores, as described in chapter 11, pp. 218 of[19], based on the nonparametric estimates of the mixture density f(z) (Eq. [S6]) obtained with *locfdr*. For each of the 70 discovery sets used to calculate cumulative replication rates we calculated the expected *a posteriori* effect size across the same 120 equally sized z-score bins ranging from -5.33 to 5.33 (corresponding to the GWAS p-value of 5x10^-8^). The results were averaged across the 70 iterations and plotted as a function of discovery z-score independently for each genic annotation category. Because the direction of effect (z-score sign) is arbitrary with respect to the allele and strand chosen as causal, the data were duplicated with opposite sign to enforce symmetry. Again this procedure was carried out for the overall data and per category (Figure S10).

For comparison, empirical replication z-scores were calculated using the same 70 discovery-replication pairs and averaged across iterations. For visualization a cubic smoothing spline was fit relating the discovery z-score bin midpoints to the corresponding average replication z-scores. Our empirical z-score replications (Figure S10B) closely match the theoretical expected values (Figure S10A) and suggest that the *a posteriori* effect size for a given SNP is strongly modulated by genic annotation category.

**A Parametric Mixture Model**

In addition to the non-parametric approach to estimating the mixture model (Eq. [S6]) implemented in the *locfdr* package, we implemented a parametric model, to facilitate simulations and extensions of the basic locfdr model to include covariates, described below. Specifically, we modeled w = -2 ln(p), as a mixture of a (null) χ^2^ density with two degrees of freedom and a (non-null) Weibull density with shape parameter a and scale parameter b. Note, under the null hypothesis the p-values are uniformly distributed and hence w has a χ^2^ density with two degrees of freedom (df), equivalent to a Weibull density with a=1 and b=2. Hence, the mixture density for w is given by

f(w) = π_0_f_0_(w) + π_1_f_1_(w)*,* [S9]

where f_0_(w) is Weibull(a_0_=1, b_0_=2) and f_1_(w) is Weibull(a_1_, b_1_), where the parameters (π_0_, a_1_,b_1_) are estimated from the data. For identifiability, the model is fit under the assumption (in common with the *locfdr* package) that the non-null density is zero in a small interval around zero, accomplished here by shifting f_1_ to the right by a fixed margin, e.g., the median of the χ^2^ distribution with 2 df. This is equivalent to the assumption that the vast majority of SNPs with z-scores close to zero are true nulls[19]. For parameter estimation we used a Bayesian Monte Carlo Markov Chain (MCMC) algorithm, placing vague priors on the parameters (π_0_, a_1_,b_1_). Q-Q plots and model fits for Height and CD for SNPs below the GWAS-level significance threshold of 5 x 10^-8^ are given in Figure S17. For Height, parameter estimates from the MCMC algorithm were (π_0_, a_1_,b_1_) = (0.959, 0.8, 5.7)**;** for CD, parameter estimates were (π_0_, a_1_,b_1_) = (0.974, 0.8, 4.1)**.**

We used the CD parameter estimates to determine the impact of sample size and polygenicity on Q-Q plots and enrichment indices in the context of mixture models. Figure S18 shows the impact of polygenicity (i.e., the non-null proportion π_1_). The solid black line is the Q-Q curve for CD predicted from the Weibull mixture model, with π_1_=.0.026. The red line is the predicted Q-Q curve if π_1_ = 0.10 (more polygenic) and the blue line is the predicted Q-Q curve if π_1_ = 0.001 (less polygenic). Phenotypes that are more polygenic but otherwise have similar non-null densities f_1_ have Q-Q curves that depart earlier from the non-null line but are approximately parallel thereafter. In contrast, for a fixed level of polygenicity but varying non-null distributions, Q-Q plots tend to depart from the null line at the same place but have different slopes thereafter. This can be illustrated by varying the effective sample size of the GWAS: increasing sample size leaves π_1_ (the true proportion of non-null SNPs) fixed but increases the scale of the non-null density f_1_. Figure S19 shows the impact for decreasing or increasing the sample size on the Q-Q plots for the CD data.

We also extended the basic parametric mixture model [S9] by allowing for covariates (e.g., genic annotations). Specifically, let **x** be a vector of annotations for a given SNP. The *covariate-modulated mixture model* is given by

f(w | **x**) = π_0_(**x**)f_0_(w) + π_1_(**x**)f_1_(w| **x**)*,* [S10]

where π_0_(**x**) = 1 **/** (1 + exp(**x’ν**)) is a logistic function of the covariates, and f_1_(w| **x**) is a Weibull distribution with shape parameter a=exp(**x’α**) and scale parameter b=exp(**x’β**). We estimate the model using an MCMC algorithm (Gibbs sampler with Metropolis-Hastings steps), placing non-informative priors on unknown parameters (**ν**, **α**, **β**). Estimates from this model, not presented here, could be used to replace the stratified FDR analyses in the main text by directly using Eq. [S10] to estimate the local fdr (Eq. [S8]).

**Control for potential confounds: LD and MAF**

We report significant categorical differences in terms of total LD and total number of SNPs captured by each GWAS SNP that mirrors our enrichment findings (Tables S7 and S8). To rule out total LD as a potential confound, a multiple regression was performed on height GWAS summary values (log of z^2^ after intergenic inflation control) using SNP annotation category scores and total summed LD as predictors. Each category score is computed as described in the main text. The category score of each SNP is pre-multiplied by the genetic variance (MAF*(1-MAF)) of that SNP. Annotations categories were centered to have mean zero. Our analysis reveals only a minor effect of total LD on predicting log(z^2^) and strong individual category effects that mirror our enrichment findings (Table S10).

Systematic differences in the average minor allele frequency (MAF) could confound enrichment analysis as MAF acts multiplicatively with effect size to give z-scores. We present the average minor allele frequency per category and do not see a categorical stratification (Table S9). Further, we demonstrate a consistent trend across categories whereby genetic variance (MAF*(1-MAF)) is proportional to variance in z-scores for height (Figure S6). Interestingly, while this trend is consistent across the summary statistic distribution for height (Figure S7A) it appears to show a different pattern in schizophrenia (Figure S7B) where more common SNPs are enriched for smaller z-scores and rare SNPs are enriched for larger z-scores.

**Replication estimates**

The estimated TDR can be thought of as the replication rate in an independent sample as the replication sample size goes to infinity. In practice, both the estimated TDR and the replication sample effect sizes will be measured with error, and hence the estimated TDR will not perfectly predict the independent sample replication rate. Nonetheless, there should be a close correspondence for reasonable discovery and replication sample sizes. Thus, to provide empirical support for our findings we explored category-specific rates of replication across eight truly independent GWAS samples studying CD. For each of eight sub-studies contributing to the final meta-analysis in the CD report we adjusted the reported z-scores according to our intergenic inflation correction method described above. For each of the 70 (8 choose 4) possible combinations of four-study discovery and four-study replication sets, we calculated the four-study combined discovery z-score and four-study combined replication z-score for each SNP as the average z-score across the four studies, multiplied by the square root of the number of studies. For discovery samples the z-scores were converted to two-tailed p-values, while replication samples were converted to one-tailed p-values preserving the direction of effect in the discovery sample. Replication was defined as a one-tailed p-value less than .05 in the replication set. For each of the 70 discovery-replication pairs cumulative rates of replication were calculated over 1000 equally-spaced bins spanning the range of negative log_10_(p-values) observed in the discovery samples. The cumulative replication rate calculated for any bin was the total number of replicated SNPs (p < .05, one-tailed test with direction of effect given by the discovery sample) with a negative log_10_(discovery p-value) greater than or equal to the lower bound of the bin divided by the total number of SNPs with a negative log_10_ (discovery p-value) greater than or equal to the lower bound of that bin. This analysis was repeated for each of the eight genic annotation categories as well as intergenic SNPs and all SNPs. The cumulative replication rates were averaged across the 70 discovery-replication pairs and the results are reported in Figure 3. The vertical intercept is the overall replication rate.

**Robustness of LD Weighted Scoring Procedure**

The original LD weighted annotation scoring approach (see: *Linkage Disequilibrium (LD) Weighted Annotation Score* above) only considered pairwise r^2^ LD greater than 0.2 and within 1 megabase of the target GWAS SNP. However, it is likely that true correlations exist at lower level than r^2^ = 0.2 and beyond 1 megabase. To test the dependence of our results upon the parameters of our scoring approach, we reclassified each SNP, following the same procedure as before, but including estimated r^2^ LD greater than 0.05 and within 2 megabases. The pattern of enrichment described in the original stratified QQ-plots appears robust to these changes (Figure S13). Three subtle qualitative trends that did emerge in the more inclusive LD scoring across most to all traits (data not shown) were: a noticeable reduction in the enrichment of the intergenic category relative to all SNPs, a slight decrease in the enrichment of the intronic category relative to all SNPs, and a slight increase in the enrichment of the 5’UTR category relative to the exon and 3’UTR categories. Further, the quantification of enrichment as mean(z^2^-1) presented in Figure 3 and Figure S6 is likewise robust to the scoring parameters (Figure S14). As with the original LD weighted scoring parameters, the differential enrichment corresponds to a mirroring increase in replication rates across independent samples (Figure S15). In addition to choosing parameters for thresholding LD to assign LD weighted annotation scores, GWAS tag SNPs were assigned to a category according to a threshold on their total LD weighted score with 1000 SNPs of a particular variety (original threshold was 1). Figure S16 shows the relationship between the mean(z^2^) of a particular SNP category and the threshold for inclusion for height. The monotonic relationship and the different slopes among the categories show our enrichment results to be consistent across a number of thresholds. One noticeable exception in Figure S16A is that the 5’UTR category decreases its mean(z^2^) when the threshold becomes very high. There are very few SNPs that remain at this point making the line unstable. Choosing a more liberal LD weighting scheme (Figure S16B) increases the number of SNPs in this category with high scores and recovers the trend. These trends are generally consistent across all other phenotypes (data not shown). Together these results demonstrate that our results are robust to the parameters within the LD-weighted annotation scoring procedure and, in fact, would likely be strengthened by a careful tuning of these parameters.

**Supplementary References**

1. Speliotes EK, Willer CJ, Berndt SI, Monda KL, Thorleifsson G, et al. (2010) Association analyses of 249,796 individuals reveal 18 new loci associated with body mass index. Nat Genet 42: 937-948.

2. Lango Allen H, Estrada K, Lettre G, Berndt SI, Weedon MN, et al. (2010) Hundreds of variants clustered in genomic loci and biological pathways affect human height. Nature 467: 832-838.

3. Heid IM, Jackson AU, Randall JC, Winkler TW, Qi L, et al. (2010) Meta-analysis identifies 13 new loci associated with waist-hip ratio and reveals sexual dimorphism in the genetic basis of fat distribution. Nat Genet 42: 949-960.

4. Teslovich TM, Musunuru K, Smith AV, Edmondson AC, Stylianou IM, et al. (2010) Biological, clinical and population relevance of 95 loci for blood lipids. Nature 466: 707-713.

5. Ehret GB, Munroe PB, Rice KM, Bochud M, Johnson AD, et al. (2011) Genetic variants in novel pathways influence blood pressure and cardiovascular disease risk. Nature 478: 103-109.

6. Franke A, McGovern DP, Barrett JC, Wang K, Radford-Smith GL, et al. (2010) Genome-wide meta-analysis increases to 71 the number of confirmed Crohn's disease susceptibility loci. Nat Genet 42: 1118-1125.

7. Anderson CA, Boucher G, Lees CW, Franke A, D'Amato M, et al. (2011) Meta-analysis identifies 29 additional ulcerative colitis risk loci, increasing the number of confirmed associations to 47. Nat Genet 43: 246-252.

8. Schizophrenia Psychiatric Genome-Wide Association Study (GWAS) Consortium (2011) Genome-wide association study identifies five new schizophrenia loci. Nat Genet 43: 969-976.

9. Psychiatric GWAS Consortium Bipolar Disorder Working Group (2011) Large-scale genome-wide association analysis of bipolar disorder identifies a new susceptibility locus near ODZ4. Nat Genet 43: 977-983.

10. Tobacco and Genetics Consortium (2010) Genome-wide meta-analyses identify multiple loci associated with smoking behavior. Nat Genet 42: 441-447.

11. Purcell S (2009) Plink. 1.07 ed.

12. Purcell S, Neale B, Todd-Brown K, Thomas L, Ferreira MA, et al. (2007) PLINK: a tool set for whole-genome association and population-based linkage analyses. Am J Hum Genet 81: 559-575.

13. Torkamani A, Scott-Van Zeeland AA, Topol EJ, Schork NJ (2011) Annotating individual human genomes. Genomics 98: 233-241.

14. Hsu F, Kent WJ, Clawson H, Kuhn RM, Diekhans M, et al. (2006) The UCSC Known Genes. Bioinformatics 22: 1036-1046.

15. Devlin B, Roeder K (1999) Genomic control for association studies. Biometrics 55: 997-1004.

16. Yang J, Weedon MN, Purcell S, Lettre G, Estrada K, et al. (2011) Genomic inflation factors under polygenic inheritance. Eur J Hum Genet 19: 807-812.

17. Schweder T, Spjotvoll E (1982) Plots of P-Values to Evaluate Many Tests Simultaneously. Biometrika 69: 493-502.

18. Benjamini Y, Hochberg Y (1995) Controlling the False Discovery Rate: A Practical and Powerful Approach to Multiple Testing. Journal of the Royal Statistical Society Series B (Methodological): Blackwell Publishing. pp. 289-300.

19. Efron B (2010) Large-scale inference : empirical Bayes methods for estimation, testing, and prediction. Cambridge ; New York: Cambridge University Press. xii, 263 p. p.

20. Efron B (2007) Size, power and false discovery rates. The Annals of Statistics 35: 1351–1377.

21. Allison DB, Gadbury GL, Heo MS, Fernandez JR, Lee CK, et al. (2002) A mixture model approach for the analysis of microarray gene expression data. Computational Statistics & Data Analysis 39: 1-20.

22. Ginns EI, St Jean P, Philibert RA, Galdzicka M, Damschroder-Williams P, et al. (1998) A genome-wide search for chromosomal loci linked to mental health wellness in relatives at high risk for bipolar affective disorder among the Old Order Amish. Proc Natl Acad Sci U S A 95: 15531-15536.

23. Efron B, Turnbull BB, Narasimhan B (2011) locfdr: Computes local false discovery rates.

24. Cardno AG, Marshall EJ, Coid B, Macdonald AM, Ribchester TR, et al. (1999) Heritability estimates for psychotic disorders: the Maudsley twin psychosis series. Arch Gen Psychiatry 56: 162-168.

25. Maes HH, Neale MC, Eaves LJ (1997) Genetic and environmental factors in relative body weight and human adiposity. Behav Genet 27: 325-351.

26. Ahmad T, Satsangi J, McGovern D, Bunce M, Jewell DP (2001) Review article: the genetics of inflammatory bowel disease. Aliment Pharmacol Ther 15: 731-748.

27. Rose RJ, Broms U, Korhonen T, Dick DM, Kapiro J (2009) Genetics of Smoking Behavior. In: Kim Y-K, editor. Handbook of Behavior Genetics. 1 ed. New York: Springer. pp. 411-432.

28. Kathiresan S, Manning AK, Demissie S, D'Agostino RB, Surti A, et al. (2007) A genome-wide association study for blood lipid phenotypes in the Framingham Heart Study. BMC Med Genet 8 Suppl 1: S17.

29. Visscher PM, Medland SE, Ferreira MA, Morley KI, Zhu G, et al. (2006) Assumption-free estimation of heritability from genome-wide identity-by-descent sharing between full siblings. PLoS Genet 2: e41.

30. Sullivan PF, Kendler KS, Neale MC (2003) Schizophrenia as a complex trait: evidence from a meta-analysis of twin studies. Arch Gen Psychiatry 60: 1187-1192.

31. Sun L, Craiu RV, Paterson AD, Bull SB (2006) Stratified false discovery control for large-scale hypothesis testing with application to genome-wide association studies. Genet Epidemiol 30: 519-530.
